# Phytonutrients and outcomes following breast cancer: a systematic review and meta-analysis of observational studies

**DOI:** 10.1093/jncics/pkad104

**Published:** 2023-12-09

**Authors:** M Diana van Die, Kerry M Bone, Kala Visvanathan, Cecile Kyrø, Dagfinn Aune, Carolyn Ee, Channing J Paller

**Affiliations:** NICM Health Research Institute, Western Sydney University, Penrith, NSW, Australia; Integria (MediHerb), Warwick, QLD, Australia; Northeast College of Health Sciences, Seneca Falls, NY, USA; Department of Oncology, The Sidney Kimmel Comprehensive Cancer Center at Johns Hopkins Medicine, Baltimore, MD, USA; Department of Epidemiology, Johns Hopkins University Bloomberg School of Public Health, Baltimore, MD, USA; Department of Diet, Cancer and Health, Danish Cancer Institute, Danish Cancer Society, Copenhagen, Denmark; Department of Epidemiology and Biostatistics, School of Public Health, Imperial College London, London, UK; Department of Nutrition, Oslo New University College, Oslo, Norway; Department of Research, The Cancer Registry of Norway, Oslo, Norway; NICM Health Research Institute, Western Sydney University, Penrith, NSW, Australia; Department of Oncology, The Sidney Kimmel Comprehensive Cancer Center at Johns Hopkins Medicine, Baltimore, MD, USA

## Abstract

**Background:**

Phytonutrient intakes may improve outcomes following breast cancer, but the impact of postdiagnosis introduction vs established prediagnostic exposure as well as optimum doses has not been established. Evidence from observational studies for key exposures was evaluated, including dosage and intake time frames.

**Methods:**

MEDLINE, EMBASE, CINAHL, Cochrane Library, ClinicalTrials.gov, and the ISRCTN registry were searched for prospective and retrospective observational studies investigating the impact of soybean, lignans, cruciferous (cabbage-family) vegetables, green tea, or their phytonutrients on breast cancer survival outcomes. A random-effects model was used to calculate summary hazard ratios (HRs) and 95% confidence intervals (CIs). Nonlinear dose-response analyses were conducted using restricted cubic splines.

**Results:**

Thirty-two articles were included. Soy isoflavones were associated with a 26% reduced risk of recurrence (HR = 0.74, 95% CI = 0.60 to 0.92), particularly among postmenopausal (HR = 0.72, 95% CI = 0.55 to 0.94) and estrogen receptor–positive survivors (HR = 0.82, 95% CI = 0.70 to 0.97), with the greatest risk reduction at 60 mg/day. In mortality outcomes, the reduction was mostly at 20 to 40 mg/day. Soy protein and products were inversely associated with cancer-specific mortality for estrogen receptor–positive disease (HR = 0.75, 95% CI = 0.60 to 0.92). An inverse association was observed for serum or plasma enterolactone, measured prediagnosis and early postdiagnosis, with cancer-specific mortality (HR = 0.72, 95% CI = 0.58 to 0.90) and all-cause mortality (HR = 0.69, 95% CI = 0.57 to 0.83). No effects were observed for cruciferous vegetables. There was a 44% reduced risk of recurrence with prediagnostic green tea for stage I and II breast cancer (HR = 0.56, 95% CI = 0.38 to 0.83).

**Conclusions:**

Soy, enterolactone, and green tea demonstrated significant risk reductions in outcomes following breast cancer. Evidence is needed regarding the impact of postdiagnostic introduction or substantial increase of these exposures.

Phytoestrogen consumption and phytotherapy (herbal medicine, botanical medicine) use are prevalent following a breast cancer diagnosis (1-3) for reasons that include preventing recurrence or metastasis and prolonging life (4,5). Exposure to phytonutrients (bioactive phytochemicals, or plant-derived compounds) and their sources may be through established dietary habits before diagnosis, dietary modification, or phytotherapeutic supplements following diagnosis. It is therefore important to understand whether postdiagnosis initiation or established intake before diagnosis affect prognosis.

Epidemiological evidence suggests a preventative role in breast cancer for phytonutrients from soybean (*Glycine max*, [L] Merr, Fabaceae family) (6) and flaxseed, also known as linseed (*Linum usitatissimum*, Linn, Linaceae family) (7,8), Cruciferae (Brassicaceae)-family vegetables (9) (eg, broccoli, cabbage, Brussels sprouts, cauliflower, kale, kohlrabi, bok choy), and green tea (*Camellia sinensis* (L), Theaceae family) (10). Levels similar to dietary exposure can be achieved in supplemental doses prescribed by practitioners in the Anglo-American and European traditions of phytotherapy.

A growing body of evidence from in vitro, in vivo, and human biomarker studies supports the anticancer effects of several of these plants’ phytochemicals, which include phytoestrogens such as genistein and daidzein from soy and enterolactone (ENL) and enterodiol metabolized from phytoestrogenic lignans (found in whole grains, legumes, and some fruits and vegetables), notably secoisolariciresinol diglucoside (SDG) in flaxseed. Isothiocyanates and indoles hydrolyzed from glucosinolates in cruciferous vegetables, such as benzyl isothiocyanate, sulforaphane, and 3,30-diindolylmethane from indole-3-carbinol (I3C), and the green tea polyphenol epigallocatechin-3-gallate (EGCG), also have demonstrated marked protective activity. These agents can regulate several key molecular and metabolic processes identified as cancer hallmarks, including cell signaling, cell cycle regulation, oxidative stress response, inflammation, apoptosis, angiogenesis, and metastasis (11,12), thereby potentially improving cancer outcomes. Genistein, SDG, and sulforaphane additionally increase the cytotoxic effects of chemotherapeutic agents in some breast cancer cell lines, while green tea polyphenols may inhibit cancer cell resistance to chemotherapeutic drugs (12,13).The full range of their anticancer mechanisms has been extensively reviewed elsewhere (11,12).

Emerging evidence also suggests a role in improving breast cancer prognosis. Human biomarker studies show improved prognosis with prediagnosis phytoestrogen intake (14) and lignan intake in postmenopausal breast cancer. Higher lignan intake has been associated with a reduced likelihood of development of advanced breast tumors (15,16).

Observational studies have produced conflicting findings for intakes of these phytonutrients and their sources, as have meta-analyses, because of lack of consistency in study inclusion, classification of study outcomes, and exposure assessment time frame. Benefits have variously been found in risk of recurrence for soy (17-19) and green tea (20,21); breast cancer–specific mortality for soy foods (17-19) and ENL, or combined ENL and lignans (18,22,23); and all-cause mortality with soy exposure (18,19), ENL, or combined ENL and lignans (18,22). It is unclear, however, whether initiating or significantly increasing intake following diagnosis has the same effect on outcomes as established prediagnostic exposure.

The most effective dosages are not necessarily the highest intakes. Meta-analyses typically compare intakes for the highest with the lowest quantiles, which vary across study populations. This approach may not provide clinically meaningful information regarding optimal dosage levels.

The objective of this systematic review and meta-analysis was to inform clinicians, patients, researchers, and policy makers of the evidence from observational studies for soybean, lignans, cruciferous vegetables, and green tea as well as their phytonutrients on breast cancer recurrence, breast cancer–specific mortality, and all-cause mortality, with a focus on the most effective dosage and time frame for consuming these exposures.

## Methods

This review was registered with PROSPERO (CRD42022366097) and Open Science Framework and is reported according to Preferred Reporting Items for Systematic Reviews and Meta-analyses guidelines.

The following electronic databases and registries were searched (earliest to October 17, 2022; updated October 30, 2023): MEDLINE (Ovid), EMBASE (Ovid), CINAHL (EBSCO), Cochrane Library, ClinicalTrials.gov (https://clinicaltrials.gov), and the ISRCTN registry. Further relevant papers were identified by manually searching reference lists of relevant journal articles and conference proceedings.

### Search strategy

We used the following search strategy in MEDLINE, adapted for other databases:

1. Breast Neoplasms/
2. (breast adj4 (cancer or malignancy or neoplasm$or tumo? r$or carcinoma)).ti, ab.
3. 1 or 2
4. Phytotherapy/or Plant Extracts/or Plants, Medicinal/or Pharmacognosy/
5. (herb$or phytonutrient$or phytochemical$or nutraceutic$or plant$, medicinal or plant extract$or *Glycine max* or soy or isoflavone or flavonoid or phytoestrogen or genistein or daidzein or *Linum* or flaxseed or linseed or lignan$or secoisolariciresinol or SDG or *Camellia sinensis* or green tea or EGCG or epigallocatechin-3-gallate or catechin$or *Brassica*$or broccoli or indole-3-carbinol or 3,3'-diindolylmethane or sulforaphane or cabbage or cruciferous or *Cruciferae*).ti, ab.
6. 4 or 5
7. mortality/or survival rate/or neoplasm invasiveness/or neoplasm metastasis/or extranodal extension/or neoplasm recurrence, local/
8. (survival or recurrence or relapse or progression or prognosis or mortality or death).ti, ab.
9. 7 or 8
10. observational study/or epidemiologic studies/or retrospective studies/or cohort studies/or follow-up/or longitudinal studies/or prospective studies/
11. (observational study or epidemiological or cohort study or prospective or retrospective or follow-up).ti, ab.
12. 10 or 11
13. 3 and 6 and 9 and 12
14. limit 13 to humans

English-language restriction was imposed due to funding limitations.

### Study selection

We included observational studies, both prospective and retrospective.

### Types of participants

Women after treatment or currently undergoing treatment for histologically confirmed breast cancer were included.

### Types of intervention

Studies investigating the phytonutrients, soy, flaxseed (linseed), Cruciferae-family vegetables, green tea, or biomarkers of their intake were included. Studies investigating diets, multicomponent, or lifestyle interventions were excluded. Exposure was measured prediagnosis or postdiagnosis.

### Types of outcome measures

The primary outcomes were

- recurrence (death censored);
- disease-free survival (DFS; recurrence and breast cancer death);
- distant DFS;
- breast cancer mortality; and
- all-cause mortality.

### Data extraction

#### Methodologic quality assessment

Two reviewers (D.v.D. and K.B.) independently screened the titles and abstracts of all articles returned from the Covidence search. When necessary, full-text articles were obtained to determine eligibility for the study. Any disagreement between the 2 authors was resolved by a third author (C.P.). Data extraction from included studies was conducted by 2 investigators (D.v.D. and C.K.). The following data were extracted from the included articles, using a standardized extraction form in Microsoft Excel: 1) first author, publication year, and study name and country; 2) recruitment dates, duration, and follow-up; 3) study design and setting, identification of cases; 4) population characteristics; 5) exposure time frame (pre- or postdiagnosis) and exposure measurement and time frame; 6) ascertainment of events (not shown); 7) dosage ranges, overall and per quantile; 8) cohort size and number of events per quantile; 9) hazard ratios (HRs) and 95% confidence intervals (CIs); and 10) covariate adjustments. Additional data or further details were sought from 11 authors.

Studies were assessed for quality and risk of bias using a slightly modified version of the Newcastle-Ottawa Scale for nonrandomized cohort studies, which awards a possible total of 9 points based on cohort selection (4 possible points), comparability of cohorts (2 possible points), and adequacy of outcome measures (3 possible points) (24). The “Ascertainment of Exposure” criterion was adapted for food frequency questionnaires and diet diaries; the “Comparability” criterion required 5 confounders (estrogen receptor and progesterone receptor status, stage/local or distant dissemination, treatment type, age, and menopausal status) for 2 points; and adequate follow-up time was set at a minimum of 2 years for all outcomes. We assigned a rating of high quality to studies scoring 8 to 9 and moderate quality to those scoring 5 to 7.

Strength of evidence was assessed (by D.v.D., K.B., C.K,) using the adaptation by the World Cancer Research Fund International (WCRF)/American Institute for Cancer Research (AICR) of the Grading of Recommendations, Assessment, Development, and Evaluation (GRADE) (25) criteria for breast cancer survivors (26), with disagreements resolved by a third person (K.V.). The criteria lead to 5 possible levels of conclusion: 1) convincingly causal, 2) probably causal, 3) limited evidence but suggestive of a possible causal relationship, 4) limited evidence and no conclusion of a causal relationship possible, and 5) substantial effect on risk (of outcome) unlikely. The criteria allow for flexibility through specified upgrading or downgrading factors—characteristics of the evidence that strengthen or weaken confidence in an association being causal.

### Data analysis

Different units of measurement were standardized using the following conversions: for soy isoflavones and products, milligrams per 1000 kcal × 1.77 = mg/day; for cruciferous vegetables, 1 cup = 156 g, 1 serving = 78 g; and for studies based in China or Japan, 2 g = 1 cup green tea.

We used the random effects model by DerSimonian and Laird (27), which takes into account heterogeneity within and between studies, to calculate summary hazard ratios and 95% confidence intervals for the association among soy isoflavones, soy protein and products, dietary lignans, serum or plasma ENL, cruciferous vegetables, and green tea and disease outcomes (recurrence, breast cancer–specific mortality, and all-cause mortality). The method of Greenland and Longnecker (28) was used to estimate study-specific slopes (linear trends) and calculate 95% confidence intervals from the natural logarithm of the hazard ratios across categories of the selected exposures. For studies reporting means or medians per category, these estimates were used directly; if ranges were reported, the mean of the upper and lower range was calculated. For studies reporting open-ended extreme categories, the width of the adjacent category was used to estimate a lower or upper cutoff point for the open-ended categories. Nonlinear dose-response analyses were conducted to examine the shape of the dose-response relationship between the exposures and disease outcomes, using restricted cubic splines with 3 knots at 10, 50, and 90 percentiles of the distribution, which were combined using multivariable meta-analysis (29).

Heterogeneity between studies was examined using the Cochrane *Q* statistic and *I*^2^ statistic (30). The level of significance for the Cochrane *Q* test is *P *less than* *.10. (*P *<* *.10 represents statistically significant heterogeneity.) The *I^2^* statistic represents the total variation among studies that could be attributed to between-study heterogeneity. *I^2^* values of 25% or less represent no significant heterogeneity, and 25% to 50%, 50% to 75%, and greater than 75% indicated small, moderate, and significant heterogeneity, respectively. Prespecified subgroup analyses were performed to estimate the effects of menopausal status, estrogen receptor status, tumor stage, and pre- vs postdiagnosis intake.

Sensitivity analyses excluding 1 study at a time were conducted to assess the robustness of the summary estimates when at least 7 studies were included in the analysis. Publication bias was evaluated using the Egger test and by visual inspection of the funnel plots for asymmetry when at least 7 studies were included in the analysis (31). Stata, version 13.0 (StataCorp, College Station, TX) was used for the analyses.

## Results

### Study selection

**Figure 1.**
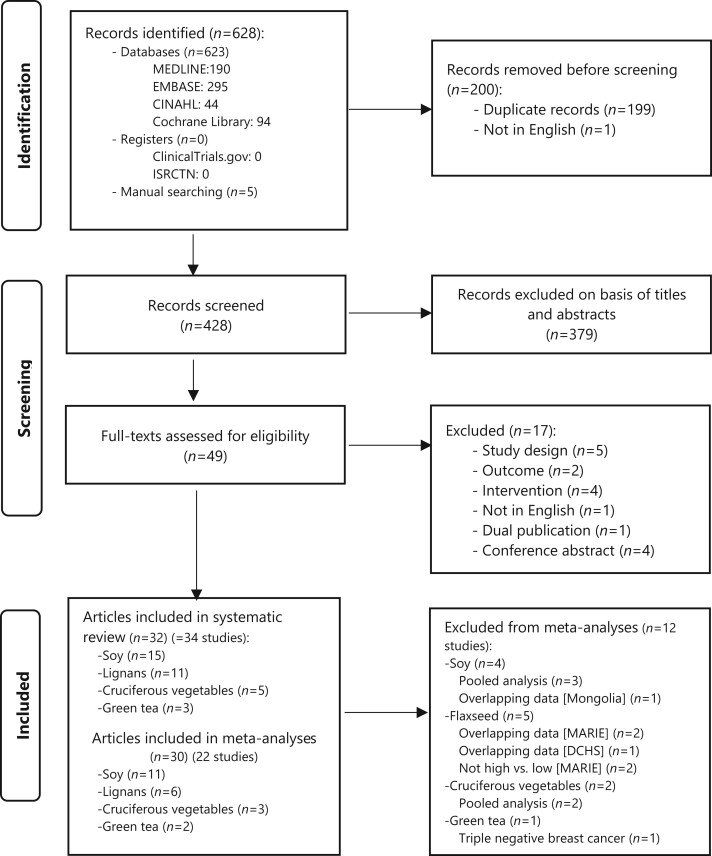
Flow diagram of the study selection process. CINAHL = the Cumulative Index of Nursing and Allied Health Literature; ISRCTN = International Standard Registered Clinical/soCial sTudy Number.

After removal of duplicate articles, 428 articles were screened and 49 full-text articles were assessed, 32 of which met eligibility criteria (Figure 1). Excluding studies with overlapping populations [5 from pooled analyses (32-36), 4 from the Mamma Carcinoma Risk Factor Investigation (MARIE) studies (15,16,37,38), 1 from Danish Diet and Health (39)], and 1 investigating only triple-negative breast cancer (40), 22 of 34 studies remained for inclusion in meta-analyses (Figure 1): 11 examined soy isoflavones (N* *=* *34 567) (41-50), protein (42,48,51), or products (N* *=* *9480) (42,48,51); 3 lignans (N* *=* *14 114) (43,46,52); 3 ENL (N* *=* *3864) (22,53,54); 3 cruciferous vegetables (N* *=* *17 041) (55-57); and 2 green tea (N* *=* *1632) (58,59). These studies included pooled analyses of soy (47) and cruciferous vegetables (57). Kyrø (46) provided additional subgroup analyses for the European Prospective Investigation into Cancer and Nutrition (EPIC) study and a further 3 years of follow-up data for the Diet, Cancer and Health study on plasma ENL (54). Nine (32-35,39,47,51,54,57) of 11 authors contacted for further information or clarification responded.

### Study characteristics

Study characteristics are presented in Table 1. All were prospective cohort studies, apart from 1 retrospective study on ENL (53). Exposure was measured using a food frequency questionnaire or a variety of such questionnaires and quantitative dietary questionnaires (46), except for 1 study, which used 24-hour diet recall (51). Three studies (32,34,35) adjusted for soy supplement intake, and a fourth reported collecting information about soy protein powder supplements (60).

**Table 1.** Characteristics of cohort studies investigating intake of soy, lignans, enterolactone, green tea, and cruciferous vegetables

Author, year, study name, country	Study design	Exposure and time frame; measurement and time frame	Diagnosis; recruitment dates–study end; follow-up duration	Participant characteristics: age, y; ethnicity, No. (%); menopause status, No. (%)	Hormone receptor status, No. (%)	Tumor characteristics, No. (%)	Treatment	Highest quantile vs lowest quantile	Recurrence, HR (95% CI), *P* for trend^a^; Total cohort (analyzed)/events, No.	Breast cancer–specific mortality, HR (95% CI), *P* for trend^a^Total cohort (analyzed)/events, No.	Disease-free survival /distant disease-free survival Total cohort (analyzed)/events, No.	All-cause mortality, HR (95% CI), *P* for trend^a^_;_ Total cohort (analyzed)/events, No.	Adjusted for	Newcastle-Ottawa Scale score
Caan, 2011 (32) Women’s Healthy Eating and Living Study, USA	Population-based prospective cohort Follow-up of a multisite randomized controlled trial of a high-vegetable, low-fat diet; ancillary analysis	Soy isoflavones Postdiagnosis Food frequency questionnaire Median, 2 y (range, 2 moto 4 y)	1991-2000 to 2006 Median follow-up: 7.3 y	Age: 18-70 y at study enrolment Ethnicity: Asian: 84 (3.1) Black: 105 (3.8) Hispanic: 149 (5.5) White: 2334 (85.3) Other: 64 (2.3) Menopause status: Premenopausal: 306 (11.2) Postmenopausal or perimenopausal: 2426 (88.7) Missing: 4 (0.1)	Estrogen receptor+ or progesterone receptor+: 2144 (78.3) Estrogen receptor– or progesterone receptor–: 546 (20.0) Missing: 46 (1.7)	Stage I: 1063 (38.9) II: 1254 (45.8) III: 419 (15.3)	Tamoxifen use: Current: 1642 (60.0) Past: 174 (6.3) Never: 884 (32.2) Missing: 36 (1.5)	Isoflavones: Q4: 16.3-86.9 mg/d vs Q1: <0.7 mg/d	0.78 (0.46 to 1.31) 3088 (2736)/448	—	—	0.46 (0.2 to 1.05), *P* = .02 3088 (2736)/271	Stage, grade, estrogen and progesterone receptor status, menopause status, chemotherapy, radiation therapy, age, education, race, soy supplements, intervention group, hot flashes, tamoxifen use	9
Guha, 2009 (34) The Life After Cancer Epidemiology study, USA	Population-based prospective cohort	Soy isoflavones: daidzein, genistein, glycitein Postdiagnosis Food frequency questionnaire Mean, 23 mo (range = 11-39 mo)	1997-2000 to 2005 Mean follow-up: 6.3 y (range = 0.11-8.7y)	Age: 18-79 y at diagnosis Ethnicity: Asian: 73 (3.7) Black: 83 (4.3) Hispanic White: 107 (5.5) Non-Hispanic White: 1591 (81.4) Other: 98 (5.0) Menopause status: Premenopausal: 416 (21.3) Postmenopausal: 1268 (64.9) Unclear: 268 (13.7)	Estrogen receptor positive: 1594 (81.6) Estrogen receptor negative: 337 (17.3) Missing: 23 (1.1)	Stage: I: 925 (47.3) IIA: 646 (33.1) IIB: 321 (16.4) IIIA: 58 (3.0) Missing: 4 (0.2)	Tamoxifen use: Current: 1382 (70.7) Past: 135 (6.9) Never: 434 (22.2) Missing: 3 (0.2)	Daidzein: Q5: ≥9597 vs Q1: 0 µg/d (Q5: ≥9.60 vs Q1: 0 mg/d)	0.96 (0.52 to 1.76) 1954 (1838)/282	—	—	—	Soy supplement use, body mass index 1 y before diagnosis, menopausal status at diagnosis, tobacco pack-years, tumor stage, estrogen receptor or progesterone receptor status, age at diagnosis, race, total energy intake	8
								Genistein: Q5: ≥13 026 vs Q1: <0.10 µg/d (Q5: ≥13.03 vs Q1: 0 mg/d)	0.95 (0.52 to 1.75) 1954 (1838)/282					
								Glycitein: Q5: ≥795.4 vs Q1: ≤3.61 µg/d (Q5: ≥0.80 vs Q1: 0.0036 mg/d)	0.80 (0.42 to 1.50) 1954 (1838)/282					
Shu, 2009 (35) Shanghai Breast Cancer Survival Study, China	Population-based prospective cohort	Soy isoflavones and soy protein Postdiagnosis Food frequency questionnaire 6.5 mo (3-11 mo), 18, 36, and 60 mo	2002-2006 to 2009 Median follow-up: 3.9 y (0.5-6.2y)	Age: 25-70 y at diagnosis Ethnicity: Asian: 5.033 (100) Menopause status: Premenopausal: 2461 (48.9) Postmenopausal: 2572 (51.1)	Estrogen receptor positive: 3181 (63.2) Estrogen receptor negative: 1772 (35.2) Missing: 80 (1.6)	Stage: 0: 156 (3.1) I: 1678 (33.4) II: 2482 (49.3) III-IV: 492 (9.8) Unknown: 223 (4.4)	Chemotherapy: Yes: 4589 (91.2) No: 444 (8.8) Radiation therapy: Yes: 1615 (32.1) No: 3418 (67.9) Radical mastectomy: Yes: 4660 (92.6) No: 373 (7.4) Tamoxifen use: Yes: 2622 (52.1) No: 2408 (47.8)	Isoflavones: Q4: >62.68 mg/d vs Q1: <20 mg/d	—	—	0.77 (0.6 to 0.98) 5033 (5032)/534	0.76 (0.58 to 0.99) 5033 (5032)/444	Age at diagnosis, TNM stage, chemotherapy, radiation therapy, type of surgery received, body mass index, estrogen receptor and progesterone receptor status, tamoxifen use, education level, crucifer intake, red meat intake, fish intake, any vitamin supplement use, tea consumption, physical activity	9
								Soy protein: Q4: >15.31 vs Q1: <5.31 g/d	—	—	0.68 (0.54 to 0.87) 5033 (5032)/534	0.71 (0.54 to 0.92) 5033 (5032)/444		
Nechuta, 2012 (47) Pooled analysis of the Women’s Healthy Eating and Living study, the Life After Cancer Epidemiology study, and the Shanghai Breast Cancer Survival Study USA and China	Population-based prospective cohort	Soy isoflavones Postdiagnosis Food frequency questionnaire Means: 23.4 mo; 22.5 mo; 6.5 mo (3-48 mo)	1995-2006 to 2006-2009 Mean follow-up: 7.4 y	Age: 18-79 y at diagnosis Ethnicity: Asian: 5056 (53.0) Black: 191 (2.0) Hispanic: 263 (2.8) Non-Hispanic White: 3902 (41.1) Other: 102 (1.1) Menopause status: Premenopausal: 4171 (43.8) Postmenopausal: 4995 (52.5) Missing or unclear: 348 (3.7)	Estrogen receptor positive: 6661 (70.0) Estrogen receptor negative: 2692 (28.3) Missing: 161 (1.7)	Stage: I: 3654 (38.4) II: 4264 (44.8) III: 1366 (14.4) Missing: 230 (2.4)	Chemotherapy: Yes: 7487 (78.7) No: 2027 (21.3) Radiation therapy: Yes: 4489 (47.2) No: 5025 (52.8) Endocrine therapy: Yes: 5962 62.7) No: 3536 (37.2) Missing: 16 (0.1) Tamoxifen use among women with estrogen receptor–positive women cancer: Yes: 5178 (77.5) No: 1505 (22.5)	Isoflavones: T3: ≥10 vs T1: <4 mg/d	0.75 (0.61 to 0.92) 9514/1348	0.83 (0.64 to 1.07) 9514/881	—	0.87 (0.70 to 1.10) 9514/1171	Age at diagnosis, estrogen and progesterone receptor status, TNM stage, chemotherapy, radiation therapy, hormone therapy, smoking status, body mass index, exercise, cruciferous vegetable intake, parity, menopause status, study, race/ethnicity (where applicable), education level	8
								D10: ≥92.6 vs D1: 9.4 mg/d	0.64 (0.50 to 0.82) 9514/1348	0.71 (0.52 to 0.97) 9514/881	—	0.82 (0.63 to 1.08) 9514/1171		
Fink, 2007 (43) Long Island Breast Cancer Study, USA	Population-based prospective cohort Follow-up of parent case-control study	Soy isoflavones Prediagnosis Food frequency questionnaire Shortly after diagnosis, intake in the previous 12 mo	1996-1997 to 2002 Follow-up: up to 8 y	Age: 25-98 y at diagnosis Ethnicity: Black: 50 (4.2) White: 1138 (94.1) Other: 21 (1.7) Missing: 1 (0.01) Menopause status: Premenopausal: 376 (31.1) Postmenopausal: 834 (68.9)	Estrogen receptor positive: 664 (54.8) Estrogen receptor negative: 241 (19.9)	NR	NR	Isoflavones: Q5: ≥7.48 vs Q1: ≤0.29 mg/d	—	0.87 (0.54 to 1.41) 1148/113	—	0.52 (0.33 to 0.82) 1210/173	Menopausal status at diagnosis, family history of breast cancer in a first-degree relative, physical activity level from menarche to diagnosis, cigarette smoking status, body mass index at diagnosis, average lifetime alcohol intake, education level, income, hormone replacement therapy, comorbidities (history of hypertension, diabetes, high cholesterol, myocardial infarction, stroke)	8
Boyapati, 2005 (41) Shanghai Breast Cancer Study, China	Population-based prospective cohort Follow-up of case-control study	Soy isoflavones and soy protein Prediagnosis Food frequency questionnaire Study entry and after 7 mo, assessed usual diet over past 5 y	1996-1998 to 2002 Median follow-up: 5.2 y	Age: 25-64y at diagnosis Ethnicity: Asian: 1459 (100) Menopause status: Prediagnosis: 951 (65.2) Postdiagnosis: 457 (31.3) Missing: 51 (3.5)	Estrogen receptor positive: 637 (43.7) Estrogen receptor negative: 364 (25) Missing: 458 (31.4)	Stage: I-II: 867 (59.4) III-IV: 114 (7.8) Unknown: 74 (5.1)	Chemotherapy: Yes: 985 (67.5) No: 58 (4.0) Unknown: 12 (0.8) Radiation therapy: Yes: 403 (27.6) No: 500 (34.3) Unknown: 152 (10.4) Surgery: Yes: 1048 (71.9) No: 1 (0.07) Unknown: 6 (0.4)	Isoflavones: T3 vs T1 (tertiles not specified)	—	1.06 (0.79to 1.42) 1459/296	—	—	Age at diagnosis, disease stage, radiation therapy, estrogen and progesterone receptor status, total energy intake	8
								Soy protein: T3 vs T1 (tertiles not specified)		0.99 (0.73 to 1.33) 1459/297	—			
Kang, 2010 (45) Harbin Medical University, China	Hospital-based prospective cohort	Soy isoflavones Prediagnosis Food frequency questionnaire NR; assessed intake over the previous 5 y	2002-2003 to 2008 Median follow-up: 5.1 y	Age: 29-72 y at diagnosis Ethnicity: Asian: 1459 (100) Menopause status: Premenopausal: 248 (47.3) Postmenopausal: 276 (52.7)	Estrogen receptor positive: 447 (85.3) Estrogen receptor negative: 77 (14.7)	Stage: I: 64 (12.2) II: 345 (65.8) III: 115 (22.0)	Chemotherapy: Yes: 446 (85.1) No: 78 (14.9) Radiation therapy: Yes: 55 (10.5) No: 469 (89.5) Endocrine therapy: Tamoxifen: 438 (83.6) Anastrozole: 86 (16.4)	Isoflavones: Q4: >42.3 vs Q1: ≤15.2 mg/d	524/185	—	—	524/154	Age at diagnosis, TNM stage, estrogen and progesterone receptor status, chemotherapy and radiation therapy	8
									Premenopause: 0.88 (0.61 to 1.23) 248/94		—	Premenopause: 1.05 (0.78 to 1.71) 248/76		
									Postmenopause: 0.67 (0.54 to 0.85) *P* = .02 for trend 276/91		—	Postmenopause: 0.88 (0.56 to 1.24) 276/78		
Zhang, 2012 (50) Inner Mongolia Medical College, China	Hospital-based prospective cohort	Soy isoflavones and soy protein Pre-/postdiagnosis unclear Food frequency questionnaire Median: 120 d from surgery to study interview	2004-2006 to 2011 Median follow-up: 4.3 y (0.75-5 y)	Age: Mean: 45.7 y at diagnosis Ethnicity: Asian: 616 (100) Menopause status: Premenopausal: 326 (52.9) Postmenopausal: 290 (47.1)	Estrogen receptor positive: 378 (61.4) Estrogen receptor negative: 238 (38.6)	Stage: 0-II: 501 (81.3) III-IV: 115 (18.7)	Chemotherapy: Yes: 534 (86.7) No: 82 (13.3) Radiation therapy: Yes: 400 (64.9) No: 216 (35.1) Hormone therapy: Yes: 47 (7.6) No: 569 (92.4) Tamoxifen use: Yes: 350 (56.8) No: 266 (43.2)	Isoflavones: Q4: >28.83 vs Q1: ≤7.56 mg/d	—	0.62 (0.42 to 0.90) 616/79	—	—	Age, education level, smoking status, alcoholic drinks consumed, family history of cancer, menopause status, tamoxifen use, TNM stage, estrogen receptor status, chemotherapy and radiation therapy	5
								Soy protein: Q4: >13.03 vs Q1: ≤2.12 g/d		0.71 (0.52 to 0.98) 566/79	—			—
Kang, 2012 (60) Inner Mongolia Medical College, China	Hospital-based prospective cohort	Soy isoflavones and soy protein Pre-diagnosis Food frequency questionnaire following diagnosis for the 12 mo before study entry	2004-2006 to 2011 Follow-up: up to 7 y	Age: Mean: 45.7 y at diagnosis Ethnicity: Asian: 288 (100) Menopause status: Premenopausal: 107 (37.3) Postmenopausal: 181 (62.7)	Estrogen receptor positive and progesterone receptor positive: 61 (21.3) Estrogen receptor negative and progesterone receptor negative: 69 (23.8) Mixed: 158 (54.9)	Stage: I: 69 (24.0) II: 88 (30.6) III: 96 (33.3) IV: 35 (12.1)	Tamoxifen use: Ever: 206 (71.4) Never: 82 (28.6)	Isoflavones: Q4: >35.3 vs Q1: ≤8.45 mg/d	—	0.25 (0.09 to 0.54) *P* < .05 for trend 288/125	—	—	Age, education level, alcohol use, smoking status, menopause status, estrogen and progesterone status, tamoxifen use, oral contraceptive use, TNM stage	6
								Soy protein: Q4: >15.78 vs Q1: <4.55 g/d		0.38 (0.17 to 0.86) 288/125	—			
Conroy, 2013 (42) Multiethnic Cohort Study, USA	Population-based prospective cohort	Soy isoflavones and soy products Prediagnosis Food frequency questionnaire At cohort entry Mean: 6.5 y before diagnosis	1993-1996 to 2007 Mean follow-up: 6.2 y	Age: Mean: 68.8 y at diagnosis Ethnicity: African American: 748 (19.5) Caucasian: 991 (25.8) Japanese American: 1141 (29.7) Latino: 623 (16.2) Native Hawaiian: 339 (8.8) Menopause status: Postmenopausal: 3842 (100) (defined as aged >50 y)	Estrogen receptor positive and progesterone receptor positive: 1748 (45.4) Estrogen receptor negative and progesterone receptor negative: 494 (12.9) Mixed: 395 (10.3) Other and unknown: 1205 (31.4)	Local: 2713 (70.6) Regional: 960 (25.0) Distant: 108 (2.8) Unstaged: 61 (1.6)	NR	Isoflavones: T3: ≥5.5 vs T1: <2.5 mg/1000 kcal (T3: ≥10.4 vs T1: <4.3 mg/d)	—	1.01 (0.74 to 1.39) 3842/376	—	0.98 (0.79 to 1.21) 3842/804	Body mass index, age at diagnosis, ethnicity, energy intake, disease stage, hormone receptor status, treatment, cardiovascular comorbidity, history of diabetes, smoking status, years between cohort entry and diagnosis	8
								Soy products: T3: ≥3.1 vs T1: 0 g/1000 kcal (T3: ≥5 vs T1: 0 g/d)		1.03 (0.71 to 1.50) 3842/376	—	1.03 (0.81 to 1.33) 3842/804		
Kyrø, 2015 (46) European Prospective Investigation into Cancer and Nutrition, France, Germany, Greece, Italy, the Netherlands, United Kingdom, Spain, Denmark, Norway, and Sweden	Population-based prospective cohort Followed up for cancer incidence and cause-specific mortality	Soy isoflavones Prediagnosis Various food frequency questionnaires Mean: 6 y	1993-1999 to 2004-2009 Median follow-up: 6.3 y	Age: Mean: 59 y at diagnosis Ethnicity: NR Menopause status: Premenopause: 2804 (23.8) Postmenopause: 8978 (76.2)	Estrogen receptor positive: 6043 (51.3) Estrogen receptor negative: 1489 (12.6) Unknown or missing: 4250 (36.1)	In situ: 911 (7.7) Localized: 4270 (36.2) Metastatic: 310 (2.6) Metastatic regional: 1618 (13.7) Metastatic distant: 84 (0.7) Unknown: 4589 (39.0)	NR	Isoflavones: Q4: >0.08 vs Q1: ≤0.01 mg/d	—	11 782/753	—	11 782/1482	Lifestyle factors: alcohol intake, body mass index, use of hormone replacement therapy, schooling, smoking status, physical activity, estrogen receptor status, cancer stage and grading of the tumor	7
										Premenopause: 0.73 (0.42 to 1.27) 2804/186	—	Premenopause: 0.80 (0.53 to 1.23) 2804/295		
										Postmenopause: 0.98 (0.72 to 1.34) 8978/567	—	Postmenopause: 1.02 (0.83 to 1.25) 8978/1187)		
Zhang, 2017 (49) USA, Canada	Population-based prospective cohort	Soy isoflavones Prediagnosis and postdiagnosis Food frequency questionnaire Within 5 y before or after diagnosis	1996 to 2011 Median follow-up: 9.4 y	Age: Mean: 51.8 y at enrolment Ethnicity: Asian: 690 (11.1) Black: 751 (12.0) Hispanic: 1033 (16.6) Non-Hispanic White: 3647 (58.5) Other: 114 (1.8) Menopause status: Premenopausal: 3056 (49.0) Postmenopausal: 3176 (51.0)	Estrogen receptor positive: 3260 (52.3) Estrogen receptor negative: 1394 (22.4) Undetermined: 120 (1.9) Missing or unknown: 1461 (23.4)	NR	Chemotherapy: Yes: 3271 (52.5) Radiation therapy: Yes: 3634 (58.3) Surgery: Yes: 5378 (86.3) Endocrine therapy: Yes: 2862 (45.9) No: 3373 (54.1)	Isoflavones: Q4: ≥1.49 vs Q1 < 0.34 mg/d	—	—	—	0.79 (0.64 to 0.97) 6235/1224	Age, study site, total caloric intake, ethnicity, education level, fiber intake, Health Eating Index, treatment type, recreational physical activity, body mass index, alcohol consumption, smoking status, menopause status, receipt of hormone therapy	8
												Prediagnosis: 0.84 (0.66 to 1.06) 4769/963		
												Postdiagnosis: 0.65 (0.41 to 1.00) *P* = .02 for trend 1466/261		
Ho, 2021 (44) Hong Kong Breast Cancer Survival Study, Hong Kong	Hospital-based prospective cohort	Soy isoflavones Prediagnosis; early postdiagnosis Food frequency questionnaire Diet for previous 12 mo assessed at study entry and 18-mo after study entry	2011-2014 to 2018 Median follow-up: 6.03 y	Age: Mean: 51.8 y at diagnosis Ethnicity: Asian: 1460 (100) Menopause status: Premenopausal: 761 (52.1) Postmenopausal: 699 (47.9)	Estrogen receptor positive: 1044 (71.5) Estrogen receptor negative: 369 (25.3) Not assessed: 47 (3.2) HER2 negative: 1035 (70.9) HER2 positive: 372 (25.5) Equivocal or not assessed: 53 (3.6) Triple negative: Yes: 187 (12.8) No: 1231 (84.3) Not assessed: 42 (2.9)	Stage: I: 531 (36.4) II: 641 (43.9) III: 288 (19.7)	Chemotherapy: Yes: 1099 (75.3) No: 361 (24.7) Radiation therapy: Yes: 1034 (70.8) No: 426 (29.2) Endocrine therapy: Yes: 1048 (71.8) No: 412 (28.2) Tamoxifen use: Yes: 634 (43.4) No: 826 (56.6)	Isoflavones: Q4: ≥7.2 vs Q1: <1.74 mg/1000 kcal (Q4: ≥12.75 vs Q1: <3.08 mg/d)	1460/210	1460/115	—	1460/130	Age, education level, menopause status, cancer stage, comorbidities, hormone receptor status (estrogen receptor positive or negative, progesterone receptor positive or negative), HER2 positive or negative status, chemotherapy, hormone therapy, tamoxifen use, radiation therapy, smoking, alcohol consumption, body mass index, waist/hip ratio, parity	8
									Prediagnosis: 1.14 (0.72 to 1.78) 1460/137	Prediagnosis: 1.25 (0.68 to 2.30) 1460/64	—	Prediagnosis: 1.08 (0.60 to 1.93) 1460/71		
									Postdiagnosis: 1.21 (0.76 to 1.93) 1460/137	Postdiagnosis: 1.24 (0.66 to 2.32) 1460/64	—	Postdiagnosis: 1.15 (0.63 to 2.10) 1460/71		
Woo, 2012 (48) Korea	Hospital-based prospective cohort	Soy isoflavones and soy products Prediagnosis Food frequency questionnaire at first breast cancer surgery (regular intake during 12 mo before first breast cancer)	2007-2008 to ? Median follow-up: 2.7 y (0.11- 4.37 y)	Age: 25-77y Ethnicity: Asian: 339 (100) Menopause status: Premenopause or perimenopause: 207 (61.0) Postmenopausal: 132 (39.0)	HER2 negative: 256 (75.5) HER2 positive: 83 (24.5)	Stage: I: 144 (42.5) II: 148 (43.6) III: 47 (13.9)	Tamoxifen use: Yes: 195 (57.5) No: 144 (42.5)	Isoflavones: T3: ≥15.2 vs T1: <7.4 mg/d	0.56 (0.20 to 1.53) 339/25	—	—	—	Total energy intake, cancer stage, age, menopause status, alcohol intake at baseline year, herceptin use, tamoxifen use	8
								Soy products: T3: ≥65.7 g/d vs T1: <36.2 g/d	0.71 (0.28 to 1.80) 339/25		—			
Yang, 2023 (51) Korea	Hospital-based prospective cohort	Soy products, fermented Postdiagnosis Structured 24-h (or 48-h) diet recall	2011-2021 to 2022 Mean follow-up: 7.42 y	Age: Mean: 54 y Ethnicity: Asian: 606 (100) Menopause status: Postmenopausal: 340 (56.1)	Estrogen receptor positive: 420 (69.3) Estrogen receptor negative: 186 (30.7) Progesterone receptor positive: 408 (67.3) Progesterone receptor negative: 198 (32.7)	Stage: 0-II: 522 (86.1) III: 77 (12)	NR	Soy products, fermented: Q4: >28.5 vs Q1: ≤5.0 g/d	0.336 (0.14 to 0.78) *P* = .016 for trend 606/61	—	—	0.173 (0.03 to 0.76) *P* = .008 for trend 606/35	Age, alcohol consumption, smoking status, tumor size, lymph node metastasis, age at menarche	7
Fink, 2007 (43) Long Island Breast Cancer Study, USA (as above)	Population-based prospective cohort Follow-up of parent case-control study	Dietary lignans Prediagnosis Food frequency questionnaire Shortly after diagnosis, previous 12 mo diet	1996-1997 to 2002 Follow-up: up to 8 y	Age: 25-98y at diagnosis Ethnicity: Black: 50 (4.2) White: 1138 (94.1) Other: 21 (1.7) Missing: 1 (0.01) Menopause status: Premenopausal: 376 (31.1) Postmenopausal: 834 (68.9)	Estrogen receptor positive: 664 (54.8) Estrogen receptor negative: 241 (19.9)	NR	NR	Dietary lignans: Q5: ≥9 vs Q1: ≤2.2 mg/d	—	0.95 (0.60 to 1.51) 1148/113	—	1.03 (0.71 to 1.49) 1210/173	Menopause status at diagnosis; family history of breast cancer in a first-degree relative, physical activity level from menarche to diagnosis, cigarette smoking status, body mass index at diagnosis, average lifetime alcohol intake, education level, income, hormone replacement therapy, comorbidities (history of hypertension, diabetes, high cholesterol, myocardial infarction, stroke)	8
McCann, 2010 (52) Western New York Exposures and Breast Cancer Study, USA	Population-based prospective cohort Follow-up of a series of population-based case-control studies	Dietary lignans Prediagnosis Food frequency questionnaire Dietary intake 12-24 mo before diagnosis	1996-2001 to 2006 Follow-up: 0.7-10.4 y	Age: Mean: 60.1 y (35-79 y) Ethnicity: Caucasian: 1038 (92.5) Other: 84 (7.5) Menopause status: Premenopausal: 315 (28.1) Postmenopausal: 807 (71.9)	Estrogen receptor positive: 651 (58.0) Estrogen receptor negative: 175 (15.6) Unknown or not done: 296 (26.4)	Stage: 0: 143 (12.7) I: 466 (41.5) II: 302 (26.9) III: 23 (2.1) IV: 35 (3.1) Missing: 154 (13.7)	NR	Dietary lignans:	—	1122/94	—	1122/160	Age, race, total energy, stage at diagnosis, body mass index, education level	8
								Q4: >257 vs Q1: <128 µg/d (Q4: 0.26 vs Q1: 0.18 mg/d) (premenopausal)		Premenopausal: 1.84 (0.65 to 5.27) *P* = .03 for trend 315/38	—	Premenopausal: 2.14 (0.82 to 5.56) *P* = .02 for trend 315/44		
								Q4: >318 vs Q1: <155 µg/d (Q4: >0.32 vs Q1: <0.16 mg/d) (postmenopausal)		Postmenopausal: 0.29 (0.11 to 0.76) *P* = .01 for trend 807/56	—	Postmenopausal: 0.49 (0.26 to 0.91) *P* = .02 for trend 807/116		
Kyrø, 2015 (46) European Prospective Investigation into Cancer and Nutrition, 10 European countries (as above)	Population-based prospective cohort Followed-up for cancer incidence and cause-specific mortality	Dietary lignans Prediagnosis Various food frequency questionnaires or self-administered quantitative dietary questionnaires Mean: 6 y	1993-1999 to 2004-2009 Median follow-up: 6.3 y	Age: Mean: 59 y at diagnosis Ethnicity: NR Menopause status: Premenopausal: 2804 (23.8) Postmenopausal: 8978 (76.2)	Estrogen receptor positive: 6043 (51.3) Estrogen receptor negative: 1489 (12.6) Unknown or missing: 4250 (36.1)	In situ: 911 (7.7) Localized: 4270 (36.2) Metastatic: 310 (2.6) Metastatic regional: 1618 (13.7) Metastatic distant: 84 (0.7) Unknown: 4589 (39.0)	NR	Dietary lignans: Q4: >2 vs Q1: ≤1.1 mg/d	—	11 782/753	—	11 782/1482	Lifestyle and clinical factors, including alcohol consumption, body mass index, hormone replacement therapy use, schooling level, smoking status, physical activity index, cancer stage, grading of tumor, estrogen receptor status, nodal status, and tumor size	7
										Premenopausal: 1.72 (0.96 to 3.09) 2804/186		Premenopausal: 1.63 (1.03 to 2.57) *P* = .01 for trend 2804/295		
										Postmenopausal: 0.72 (0.53 to 0.98) *P* = .01 for trend 8978/567		Postmenopausal: 0.86 (0.70 to 1.06) 8978/1187		
Olsen, 2011 (39) Diet, Cancer and Health study, an associated cohort of EPIC, 10 European countries (as above)	Population-based prospective cohort Followed-up for cancer incidence and mortality	Plasma enterolactone Prediagnosis Time-resolved fluoroimmunoassay Median: 8 y	1993 to 2008 Median follow-up: 10 y	Age: Median: 60 y (50-64 y) at diagnosis Ethnicity: NR Menopause status: Postmenopausal: 424 (100)	Estrogen receptor positive: 302 (71.2) Estrogen receptor negative: 89 (21.0) Unknown: 33 (7.8)	Nodal status: N0: 237 (55.9) ≥1: 159 (37.5) Unknown: 28 (6.6)	NR	Plasma enterolactone: >20.5 nmol/L vs ≤20.5 nmol/L	—	0.56 (0.36 to 0.87) 424/80	—	0.47 (0.32 to 0.68) 424/111	Tumor grade at diagnosis, baseline levels of alcohol intake, use of hormone replacement therapy	8
Kyrø, 2018 (54) (Updated in 2022) Diet, Cancer and Health study, Denmark	Population-based prospective cohort Followed-up for cancer incidence, recurrence, and mortality	Plasma enterolactone Prediagnosis High-throughput liquid chromatography-mass spectrometry/mass spectrometry method Median: 8 y	1993-2009 to 2021 Median follow-up: 9 y	Age: Median: 64 y at diagnosis Ethnicity: NR Menopause status: Postmenopausal: 1457 (100)	Estrogen receptor positive: 1118 (76.7) Estrogen receptor negative: 253 (17.4) Missing: 86 (5.9)	Nodal status: N0: 772 (53.0) 1-3: 390 (26.8) ≥4: 216 (14.8) Missing: 79 (5.4) Tumor spread at diagnosis: Yes: 42 (2.9) No: 929 (63.8) Missing: 486 (33.3)	Endocrine treatment: Yes: 663 (45.5) No: 218 (15) Unknown: 576 (39.5)	Plasma enterolactone: Q4: ≥36.9 vs Q1: ≤9.5 nmol/L	1.05 (0.72-1.51) 1457/267	0.74 (0.55 to 0.99) *P* = .0018 for trend 1457/374	—	0.74 (0.59 to 0.94) *P* = .0009 for trend 1457/604	Smoking status at baseline, smoking intensity, schooling years, body mass index, physical activity and hormone use at baseline, estrogen receptor status, nodal status, tumor size, endocrine therapy	9
Guglielmini, 2012 (53) Italy	Hospital-based retrospective cohort Follow-up study of surgical patients	Serum enterolactone Postdiagnosis Time-resolved fluoroimmunoassay serum samples obtained usually 1 mo before surgery	1984-1991 to 2010 Median follow-up: 23 y (0.6-26.1 y)	Age: Mean: 58.5 y (31-90 y) at diagnosis Ethnicity: NR Menopause status: Premenopausal: 88 (29.3) Postmenopausal: 212 (70.7)	—	Nodal status: Positive: 115 (38.3) Negative: 157 (52.3) Missing: 28 (9.4)	Chemotherapy: Yes: 91 (30.3) No: 180 (60.0) Missing: 29 (9.7) Tamoxifen use: Yes: 51 (17.0) No: 220 (73.3) Missing: 29 (9.7)	Serum enterolactone: ≥10 nmol/L vs <10 nmol/L	—	0.83 (0.53 to 1.31) 300/112	—	0.88 (0.60 to 1.29) 300/180	Menopause status, tumor size, nodal status, adjuvant chemotherapy, adjuvant tamoxifen	8
Buck, 2011a (37) Mamma Carcinoma Risk Factor Investigation, Germany	Population-based prospective cohort Follow-up of case-control study	Estimated dietary enterolactone Prediagnosis Food frequency questionnaire Dietary habits in the 12 mo before recruitment	2001-2005 to 2009 Median follow-up: 6.4 y	Age: Median: 63 y at diagnosis (50-74 y) Ethnicity: NR Menopause status: Postmenopausal: 2653 (100)	Estrogen receptor positive and progesterone receptor positive: 1540 (58.0) Estrogen receptor negative and progesterone receptor negative: 388 (14.6) Mixed: 470 (17.7) Neoadjuvant chemotherapy: 88 (3.3) In situ: 159 (6.0) Missing: 8 (0.3) HER2 positive: 432 (16.3) HER2 negative: 1723 (64.9) Missing: 251 (9.5)	Nodal status: N0: 1628 (61.4) 1-3: 550 (20.7) 4-9: 130 (4.9) ≥10: 86 (3.2) Neoadjuvant chemotherapy: 88 (3.3) In situ: 159 (6.0) Missing: 12 (0.5)	Chemotherapy: 88 (3.3) Surgery: Breast conserving: 851 (32.1) Missing: 1463 (55.1) Ablation: 339 (12.8)	Dietary enterolactone: Q5: median, 502 µg/dvs Q1: 146 µg/d	—	0.69 (0.43 to 1.10) 2653/235	—	0.60 (0.40 to 0.89) *P* = .02 for trend 2653/321	Tumor size, nodal status, metastasis, grade, estrogen and progesterone receptor status, breast cancer detection type, diabetes, menopausal hormone therapy use at diagnosis, study center, energy intake	9
Buck, 2011 b (16) Mamma Carcinoma Risk Factor Investigation, Germany	Population-based prospective cohort Follow-up of case-control study	Enterolactone, serum Postdiagnosis Time-resolved fluoroimmunoassay Blood collection median: 101 d (2-1112 d)	2002-2005 to 2009 Median follow-up: 6.1 y (0.2-7.7 y)	Age: Median: 62.8 y (50-74 y) at diagnosis Ethnicity: NR Menopause status: Postmenopausal: 1140 (100)	Estrogen receptor positive and progesterone receptor positive: 616 (54.0) Estrogen receptor negative and progesterone receptor negative: 185 (16.2) Mixed: 203 (17.8) Neoadjuvant chemotherapy 68 (6.0) In situ: 67 (5.9) HER2 positive: 202 (17.7) HER2 negative: 756 (66.3)	Nodal status: N0: 662 (58.1) 1-3: 222 (19.5) 4-9: 64 (5.6) ≥10: 53 (4.7) Neoadjuvant chemotherapy: 68 (6.0) In situ: 67 (5.9)	Chemotherapy: Adjuvant: 513 (45.0) No: 617 (54.1) Radiation therapy: Yes: 866 (76.0) No: 272 (23.9) Surgery: Breast conserving: 797 (69.9) Ablation: 331 (29.0) Aromatase inhibitor use: Yes: 529 (46.4) No: 558 (49.0) Tamoxifen use: Yes: 682 (59.8) No: 346 (30.4) Missing: 112 (9.8)	Serum enterolactone: Q4: median, 65 vs Q1: median, 3.5 nmol/L	—	—	Distant DFS: 0.62 (0.35 to 1.09) 1140/124	0.58 (0.34 to 0.99) *P* = .04 for trend 1140/162	Age at diagnosis, tumor size, nodal status, grade, estrogen and progesterone receptor status, breast cancer detection type, diabetes, hormone replacement therapy use at diagnosis, body mass index, physical activity	9
Seibold, 2014 Mamma Carcinoma Risk Factor Investigation, Germany	Population-based prospective cohort	Serum or plasma enterolactone Postdiagnosis Time-resolved fluoroimmunoassay Blood collection median: 140 d	2001-2005 to 2009 Median follow-up: 5.4 y	Age: 50-74 y at diagnosis Ethnicity: NR Menopause status: Postmenopausal: 2182 (100)	Estrogen receptor positive and progesterone receptor status: 1273 (58.3) Estrogen receptor negative and progesterone receptor negative: 309 (14.2) Mixed: 387 (17.7) Neoadjuvant chemotherapy 78 (3.6) In situ: 134 (6.1) HER2 positive: 364 (16.7) HER2 negative: 1413 (64.8)	Nodal status: N0: 1337 (61.3) 1-3: 436 (20.0) 4-9: 116 (5.3) ≥10: 79 (3.6) Neoadjuvant chemotherapy: 78 (3.6) In situ: 134 (6.1)	Chemotherapy: Yes: 1000 (45.9) No: 1156 (53.0) Radiation therapy: Yes: 1654 (75.8) No: 506 (23.2) Surgery: Breast conserving: 1503 (68.9) Ablation: 667 (30.6) Tamoxifen use: Ever: 1431 (65.6) Never: 653 (29.9)	Serum or plasma enterolactone: Q4: median, 74.2 vs Q1: median, 3.5 nmol/L	0.77 (0.51 to 1.16) 1911 (1838)/207 (188)	0.59 (0.37 to 0.94) *P* = .02 for trend 2182 (2107)/194 (186)	Distant DFS: 0.51 (0.34 to 0.77) *P* < .01 for trend 1846/214	0.59 (0.40 to 0.87) *P* < .01 for trend 2182 (2107)/269 (254)	Tumor size, nodal status, metastases status, histologic grading, estrogen and progesterone receptor status, body mass index, radiation therapy, smoking status, physical activity, menopausal hormone therapy use, mode of detection, time between blood draw and enterolactone measurement	9
Jaskulski, 2018 (15) Mamma Carcinoma Risk Factor Investigation, Germany	Population-based prospective cohort Based on cases from a case-control study	Serum/plasma enterolactone Postdiagnosis Time-resolved fluoroimmunoassay Blood draw median: 6.9 mo after operation	2002-2005 to 2009 Median follow-up: 5.3 y (0.1-7.4y)	Age: Mean: 63.2 y (50.1-75 y) at diagnosis Ethnicity: NR Menopause status: Postmenopausal: 1718 (100)	Estrogen receptor positive and progesterone receptor positive: 1016 (58.3) Estrogen receptor negative and progesterone receptor negative: 236 (13.6) Mixed: 311 (17.9) Neoadjuvant chemotherapy: 49 (2.8) In situ (stage 0): 130 (7.5)	Affected lymph nodes: 0: 1096 (62.9) 1-3: 325 (18.7) ≥4: 143 (8.2) Neoadjuvant chemotherapy: 49 (2.8) In situ/stage 0: 130 (7.5)	Chemotherapy: Yes: 763 (43.8) No: 960 (55.1) Unknown: 20 (1.2) Radiation therapy: Yes: 1342 (77.6) No: 387 (22.4) Surgery: Breast conserving: 1225 (70.3) Ablation: 518 (29.7) Tamoxifen use: Yes: 1153 (68.8) No: 523 (31.2)	Doubling in enterolactone concentration	—	0.91 (0.84 to 0.99) *P* = .02 for trend 1718/120	Distant DFS: 0.92 (0.87 to 0.99) *P* = .02 for trend 1549/181	0.93 (0.87 to 0.99) *P* = .03 for trend 1718/177	Age at diagnosis, tumor size, nodal status, histologic grading, estrogen and progesterone receptor status, mode of detection, time between operation and blood draw, body mass index, hormone replacement therapy use at diagnosis, study center	9
Jaskulski, 2020 (38) Mamma Carcinoma Risk Factor Investigation, Germany	Population-based prospective cohort Based on cases from a case-control study	Serum enterolactone Postdiagnosis Time-resolved fluoroimmunoassay Blood draw median: 4.6 mo (baseline) and 6.1 y (follow-up 1)	2002-2005 to 2015 Median follow-up: 6.4 y (median: 5.8 y [0.3-6.1y] to event censoring)	Age: Median: 63.1 y (50-74 y) at diagnosis Ethnicity: NR Menopause status: Postmenopausal: 1686 (100)	Estrogen receptor positive and progesterone receptor positive: 1021 (60.6) Estrogen receptor negative and progesterone receptor negative: 230 (13.7) Mixed: 293 (17.4) Neoadjuvant chemotherapy: 40 (2.4) In situ (stage 0): 102 (6.0) HER2 positive: 288 (17.1) HER2 negative: 1182 (70.1) Unknown: 74 (4.4)	Affected lymph nodes: 0: 1114 (66.1) 1-3: 318 (18.9) ≥4: 112 (6.6) Neoadjuvant chemotherapy: 40 (2.4) In situ (stage 0): 102 (65.0)	Chemotherapy: Yes: 677 (40.1) Neoadjuvant: 40 (2.4) No: 969 (57.5) Radiation therapy: Yes: 1368 (81.1) No: 317 (18.8) Missing: 1 (0.1) Surgery: Breast conserving: 1267 (75.2) Missing: 2 (0.1) Ablation: 417 (24.7) Tamoxifen or aromatase inhibitor use: Yes: 1364 (80.9) No: 309 (18.3) Missing: 13 (0.8)	Doubling in serum enterolactone concentration	1.14 (0.98 to 1.33) 1570/91	1.05 (0.87 to 1.26) 1674/73	—	0.98 (0.86 to 1.11) 1674/142	Age at diagnosis, time between blood draw at baseline and at follow-up 1, study center, tumor size, affected lymph nodes, grade, estrogen andprogesterone receptor status, mode of detection, recurrences between diagnosis and blood draw at follow-up 1	9
Nechuta, 2013 (57) Pooled analysis of the Life After Cancer Epidemiology study, the Women’s Healthy Eating and Living study, Nurses’ Health Study I, and the Shanghai Breast Cancer Survival Study, USA and China	Population-based prospective cohorts	Cruciferous vegetables Postdiagnosis Food frequency questionnaires Means: 23.4 mo, 2.5 mo, 23.8 mo, and 18.3 mo	1990 to 2009 Median follow-up: 5.3-12 y	Age: Mean: 57.8 y at diagnosis Ethnicity: NR Menopause status: NR	Estrogen receptor positive: 8257 (72.5) Estrogen receptor negative: NR Missing: NR	Stages I-III	NR	Q4: ≥138 vs Q1: <10.7 g/d	1.10 (0.95 to 1.28) 11 390/1421	1.09 (0.92 to 1.30) 11 390/NR	—	0.99 (0.86 to 1.13) 11 390/1725	Age at diagnosis, estrogen and progesterone receptor status, TNM stage, chemotherapy, surgery, radiation therapy, hormone therapy, smoking status, body mass index, exercise, menopause status, race or ethnicity, education level	8
Fink, 2006 (56) Long Island Breast Cancer Study, USA	Population-based prospective cohort Follow-up of a case-control study	Cruciferous vegetables Prediagnosis Food frequency questionnaire Mean: 96 d, assessed previous 12 mo diet	1996-1997 to 2002 Follow-up: up to 7 y	Age: 25-98y Ethnicity: NR Menopause status: Premenopausal: 376 (30.5) Postmenopausal: 834 (67.5) Missing: 25 (2.0)	NR	NR	Chemotherapy: Yes: 1214 (98.3) Radiation therapy: Yes: 1217 (98.5) Endocrine therapy: Yes: 1191 (96.4)	Q5: ≥6 vs Q1: 0-1 servings/wk (Q5: ≥66.85 vs Q1: ≤11 g/d]	—	—	—	1235 (1210)/186	Age at diagnosis, dietary energy intake	8
												Premenopausal: 0.72 (0.34 to 1.54) 376/43		
												Postmenopausal: 1.07 (0.67 to 1.72) 834/132		
Beasley, 2011 (55) Collaborative Women’s Longevity Study, USA	Population-based prospective cohort Recruited from parent case-control studies	Cruciferous vegetables Postdiagnosis Food frequency questionnaire 42% completed within 5 y (1-16 y)	1988 to 2005 Mean follow-up: 5.5 y	Age: 20-79 y at diagnosis Ethnicity: NR Menopause status: Premenopausal/perimenopausal: 1011 (22.8) Postmenopausal: 3254 (73.3)	NR	Local: 3233 (72.8) Regional: 1208 (27.2)	Chemotherapy: Yes: 1417 (31.9) Radiation therapy: Yes: 2210 (49.8) Surgery: Yes: 4346 (97.9) Endocrine therapy: Yes: 2568 (57.8)	Q4: median, 0.7 vs Q1: 0.1 servings/d (Q4: median, 55 vs Q1: 7.8 g/d)	—	0.95 (0.59 to 1.54) 4441/137	—	1.02 (0.80 to 1.30) 4441/525	Factors at diagnosis: age, state of residence, menopause status, smoking status, breast cancer stage, alcohol consumption, history of hormone replacement therapy Interval between diagnosis and diet assessment, factors at follow-up: energy intake, breast cancer treatment, body mass index, physical activity	7
Farvid, 2020 (33) Nurses’ Health Study I and II, USA	Population-based prospective cohort	Cruciferous vegetables Postdiagnosis Food frequency questionnaires ≥12 mo, then every 4 y	1980-2010 to 2014 (Nurses’ Health Study) and 1991-2011 to 2015 (Nurses’ Health Study II) Mean follow-up: 11.5 y (up to 30 y)	Age: 30-55 y at enrolment Ethnicity: NR Menopause status: Premenopausal: 2339 (26.2) Postmenopausal: NR	Estrogen receptor positive: 6838 (76.6) Estrogen receptor negative: 1518 (17.0) Missing: 571 (6.4)	Stage: I: 5356 (60.0) II: 2678 (30.0) III: 893 (10.0)	Chemotherapy: Yes: 4087 (45.8) Radiation therapy: Yes: 5025 (56.6) Endocrine therapy: Yes: 6177 (69.2)	Q5: median, 0.9 vs Q1: 0.1 servings/d (Q1: median, 70 vs Q1: 7.8 g/d)	—	1.02 (0.83 to 1.24) 8927/1070	—	0.87 (0.76 to 0.99) *P* = .01 for trend 8927/2521	Age at diagnosis, calendar year of diagnosis, time from diagnosis to first food frequency questionnaire, calendar year at start of follow-up, prediagnostic body mass index, body mass index change after diagnosis, postdiagnostic: smoking status, physical activity, oral contraceptive use, alcohol consumption, aspirin use, total energy intake; prediagnostic menopause status, age at menopause, postmenopausal hormone use, race, disease stage, estrogen and progesterone receptor status, radiation therapy, chemotherapy, hormone treatment	6
Thomson, 2011 (36) Women’s Healthy Eating and Living Study, USA	Population-based prospective cohort Follow-up of a multisite randomized controlled trial of a high-vegetable, low-fat diet; ancillary analysis	Cruciferous vegetables Postdiagnosis Food frequency questionnaire Mean: 23.5 mo (1.9-2 y) (and 12 and 48 mo)	1995-2006 to ? Follow-up: 7.3 y	Age: Mean: 51.2 y (18-70 y) at random assignment Ethnicity: African American: 118 (3.8) Asian: 96 (3.1) Hispanic: 164 (5.3) Pacific Islander: 23 (0.7) White: 2627 (85.3) Other: 49 (1.6) Menopause status: Premenopausal: 349 (11.3) Postmenopausal: 2442 (79.3) Perimenopausal: 284 (9.2) Unsure: 5 (0.2)	Estrogen receptor positive: 2286 (74.2) Estrogen receptor negative: 755 (24.5)	Stage: I: 1187 (38.5) IIA: 1023 (33.2) IIB: 384 (12.5) IIIA: 372 (12.1) IIIC: 114 (3.7)	Chemotherapy: Yes: 2155 (70.0) Radiation therapy: Yes: 1893 (61.5) Tamoxifen use: Current: 1833 (59.5)	T3: ≥44.6 vs T1: <13.0 g/d	0.85 (0.69 to 1.06) 3080/516	—	—	—	Time from diagnosis to study entry, menopause status, intervention status, cancer stage, estrogen receptor status, chemotherapy, body mass index, physical activity, clinical site	8
Nakachi, 1998 (59) Japan	Hospital-based prospective cohort	Green tea Prediagnosis Standardized questionnaire NR	1984-1993 to ? Mean follow-up: 6.5 y	Age: Mean: 49.7 y Ethnicity: Asian (Japanese): 472 (100) Menopause status: Premenopausal: 287 (60.8) Postmenopausal: 163 (34.5) Perimenopausal: 22 (4.7)	NR	Stage: I: 117 (24.8) II: 273 (57.8) III: 82 (17.4)	NR	≥5 vs ≤4 cup/d (epigallocatechin-3-gallate: 30-40 mg per cup)	0.78 (0.53 to 1.13) 472/123	—	—	—	Metastasized axillary lymph nodes; progesterone receptor status; clinical staging; histologic spread; use of chemotherapy; blood vessel invasion; intake of meat, fish, eggs, milk, soybean products, soybean paste soup, nuts, seaweed, fruits, green and yellow vegetables, coffee, and black and green tea; cigarette smoking status; alcohol use; frequency of pregnancy, child birth, and abortion; body mass index	7
Inoue, 2001 (58) The Hospital-based Epidemiologic Research Program at Aichi Cancer Center, Japan	Hospital-based prospective cohort	Green tea Prediagnosis Hospital-based Epidemiologic Research Program at Aichi Cancer Center questionnaire 95% within 1 y prior	1990-1998 to 1999 Mean follow-up: 4.5 y	Age: Mean: 51.5 y (23-86 y) Ethnicity: Asian (Japanese): 1160 (100) Menopause status: Premenopausal: 560 (48.4) Postmenopausal: 538 (46.5) Perimenopausal: 58 (5.0) Unknown: 4 (0.1)	NR	Stage: I: 751 (64.7) II: 257 (22.2) III and IV: 152 (13.1)	NR	T3: ≥6 vs T1: ≤2 cups/d	0.68 (0.40 to 1.16) 1160/133	—	—	—	Stage, age, year and season at first hospital visit, family history of breast cancer, body mass index, regular physical exercise, age at menarche, age at first full-term pregnancy, parity, menopause status, tofu intake, fruit intake, coffee intake	8
								T3: ≥3 vs T1: ≤2 cups/d	0.69 (0.47 to 1.00) 1160/134					
Bao, 2015 (40) Shanghai Breast Cancer Survival study, China	Population-based prospective cohort	Green tea (89%) or black tea (11%) Postdiagnosis Food frequency questionnaire 6 mo (and 18, 36, and 60 mo)	2002-2006 to 2014 Median follow-up: 9.1 y (0.6-11.8 y)	Age: Mean: 53.4 (20-75 y) at diagnosis Ethnicity: Asian (Chinese): 518 (100) Menopause status: Premenopausal: 243 (47.0) Postmenopausal: 275 (53.0)	Triple negative: 518 (100)	Stage: I: 160 (31.0) II: 288 (56.0) III: 53 (10.0) Unknown: 17 (3.0)	Chemotherapy: Yes: 489 (94.4) No: 29 (5.6) Radiation therapy: Yes: 142 (27.0) No: 376 (73.0) Radical mastectomy: Yes: 498 (96.0) No: 23 (4.0) Tamoxifen use: Yes: 112 (22.0) No: 406 (78.0)	T3: ≥100 vs T1: 0 g/mo (T3: ≥1.64 vs T1: 0 cups/d)	—		DFS: 0.72 (0.35 to 1.47) 518/112	0.76 (0.42 to 1.36) 518/128	TNM stage, chemotherapy, radiation therapy, age at diagnosis, education level, menopause status, intake of soy protein, body mass index at baseline, comorbidities, exercise participation	8

a CI = confidence interval; DFS = disease-free survival; HR = hazard ratio; N0 = no affected lymph nodes; NR = not reported; Q4 = quartile; Q5 = quintile; TNM = tumor, node and metastasis.

Postdiagnostic exposure was measured in only 3 of the studies on soy isoflavones included in meta-analyses: 1 less than 12 months and 2 more than 12 months following diagnosis. Of the 3 plasma or serum ENL studies, measurements were prediagnostic in 1 study and less than 12 months after diagnosis in the other 2. The lignan and green tea studies were all for prediagnostic exposure assessment. One cruciferous vegetable study examined prediagnostic intake, and 2 examined postdiagnostic intake (combining <12 months, with a ≥12-month exposure assessment). The interval between exposure measurement and diagnosis ranged from shortly after diagnosis to 5 years or more (43,54).

The median follow-up duration among the soy studies was 5 to 10 years in 9 (of 11) studies and less than 5 years in the remaining 2 studies. In ENL studies, the median follow-up periods were 5.4 years, 9 years, and 23 years. For the cruciferous vegetable studies reporting medians, the range was 5.3 to 12 years. The 2 green tea studies had mean follow-up times of 4.5 and 6.5 years.

Seven of the soy studies were conducted in Asian countries, and 6 were conducted in Western countries. The lignan and ENL studies were all conducted in the United States or Europe. All cruciferous vegetable studies were conducted in the United States, apart from 1 (Shanghai Breast Cancer Survival study [SBCSS]) included in the pooled analysis. All green tea studies were conducted in Asia.

### Risk of bias

Overall, the quality of studies was high. Twenty-seven studies were assessed as high quality on the Newcastle-Ottawa Scale (Table 1), and 7 were assessed as moderate. Most neglected criteria included 1) demonstrating that the outcome of interest was not present at the start of the study and 2) adjusting or testing for essential confounders—notably, treatment type, followed by estrogen receptor and progesterone receptor status, and disease stage (Supplementary Table 1, available online).

**Figure 2.**
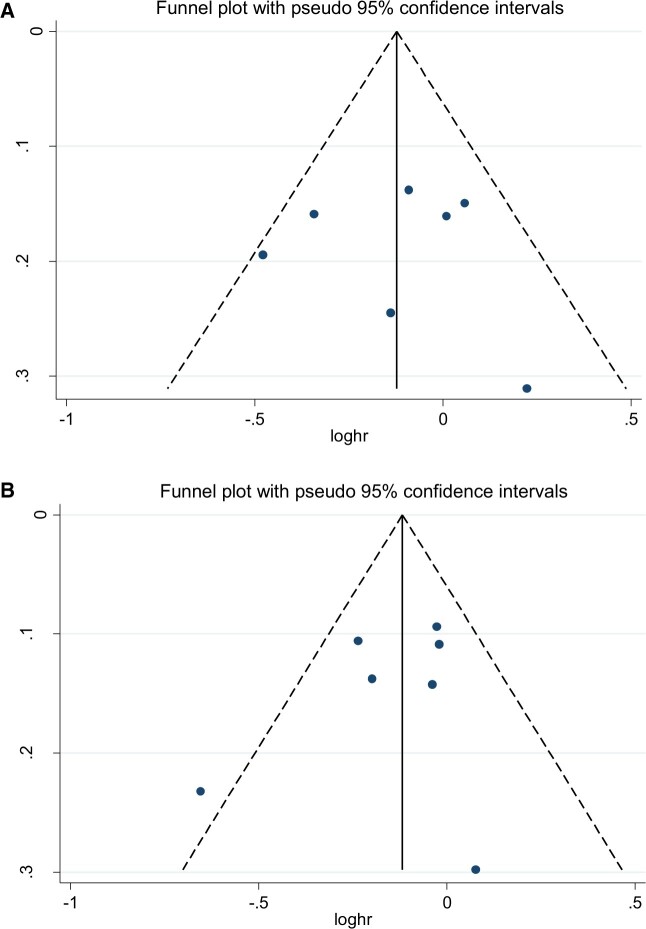
Publication bias for soy isoflavone studies on **A**) breast cancer–specific mortality, Egger: 0.92, Begg: 0.99; **B**) all-cause mortality, Egger: 0.44, Begg: 0.55.

Only for the soy isoflavones analyses were there sufficient studies to test for publication bias. No evidence of publication bias was detected in 1) breast cancer–specific mortality using the Begg test (*P* = .99) or Egger test (*P* = .92) or 2) all-cause mortality using the Begg test (*P* = .55) or the Egger test (*P* = .44) (Figure 2).

### Impact of exposures on breast cancer survival outcomes

**Figure 3.**
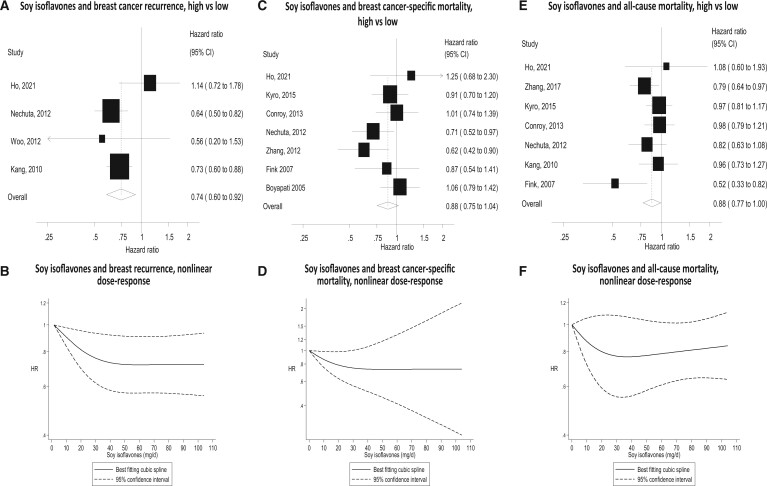
Forest plots for intake of soy isoflavones high vs low analyses and risk of recurrence, breast cancer–specific mortality, and all-cause mortality, with graphs illustrating nonlinear dose response. CI = confidence interval.

The associations between the exposures and survival outcomes from meta-analyses of the highest vs lowest intake quantiles are shown in Table 2, Figures 3 through 5, and Supplementary Figures 1 and 2 (available online).

**Table 2.** Overall and subgroup analyses of the association between soy isoflavones, protein and products, lignans, serum/plasma enterolactone concentrations, cruciferous vegetables, and green tea with breast cancer recurrence, breast cancer–specific mortality, and all-cause mortality

Exposure and outcome	Subgroup	Total subgroup cohort, No.	Studies, No.	High vs low analysis		
				Hazard ratio (95% confidence interval)	*I* ^2^ (%)	*P* for heterogeneity
Soy isoflavones						
Recurrence	Overall	11 837	4	0.74 (0.60 to 0.92)^a^	58.3	.07
	Prediagnosis	2323	3	0.82 (0.58 to 1.15)	44.9	.16
	Postdiagnosis	10 974	2	0.85 (0.46 to 1.59)	82.1	.02
	Premenopausal	5179	3	0.92 (0.74 to 1.14)	0	.94
	Postmenopausal	5971	3	0.72 (0.55 to 0.94)^a^	45.8	.16
	Estrogen receptor positive	8152	3	0.82 (0.70 to 0.97)^a^	0	.67
	Estrogen receptor negative	3139	3	0.93 (0.60 to 1.44)	61.7	.07
	Tamoxifen	6249	3	0.79 (0.55 to 1.14)	50.7	.13
	No tamoxifen	2333	2	1.02 (0.57 to 1.84)	66.5	.08
Breast cancer–specific mortality	Overall	29 817	7	0.88 (0.75 to 1.04)	30.9	.19
	Prediagnosis	19 975	7	0.92 (0.73 to 1.16)	51.0	.07
	Postdiagnosis	10 974	2	0.88 (0.52 to 1.49)	58.8	.12
	Premenopausal	7342	3	0.90 (0.67 to 1.21)	0	.67
	Postmenopausal	29 817	3	0.92 (0.77 to 1.10)	0	.69
	Estrogen receptor positive	14 260	4	0.81 (0.65 to 1.02)	15.0	.32
	Estrogen receptor negative	4913	4	0.77 (0.60 to 0.98)^a^	0	.81
All-cause mortality	Overall	34 567	7	0.88 (0.77 to 0.997)^a^	36.1	.15
	Prediagnosis	23 587	6	0.88 (0.75 to 1.04)	29.8	.21
	Postdiagnosis	12 440	3	0.81 (0.64 to 1.03)	11.0	.33
	Premenopausal	11 415	6	0.96 (0.81 to 1.14)	0	.78
	Postmenopausal	22 801	7	0.88 (0.76 to 1.02)	29.5	.20
	Estrogen receptor positive	15 496	4	0.96 (0.79 to 1.16)	0	.95
	Estrogen receptor negative	6212	4	0.93 (0.72 to 1.22)	0	.82
	Stages I-II	5334	2	1.22 (0.68 to 2.18)	0	.39
	Stages III-IV	780	2	0.58 (0.39 to 0.87)^a^	0	.43
	Tamoxifen	8673	3	0.81 (0.64 to 1.01)	0	.51
	No tamoxifen	5705	3	0.78 (0.62 to 0.98)^a^	4.4	.35
Soy protein and products						
Recurrence (products)	Overall	945	2	0.48 (0.23-0.99)^a^	25.4	.25
Breast cancer–specific mortality	Overall	5866	3	0.92 (0.76-1.11)	0	.41
	Premenopausal	951	1	1.09 (0.74-1.60)	—^c^	—^c^
	Postmenopausal	4299	2	0.93 (0.69 to 1.25)	0	.39
	Estrogen receptor positive	2604	3	0.75 (0.60 to 0.92)^a^	0	.90
	Estrogen receptor negative	991	3	0.93 (0.49 to 1.78)	64.2	.06
All-cause mortality	Overall	9480	3	0.77 (0.49 to 1.21)	74.3	.02
	Estrogen receptor positive	4928	2	0.78 (0.50 to 1.21)	59.1	.12
	Estrogen receptor negative	2266	2	1.10 (0.51 to 2.37)	76.3	.04
Lignans						
Breast cancer–specific mortality	Overall	14 052	3	0.91 (0.74 to 1.12)	55.8	.10
	Premenopausal	3486	3	1.55 (1.01 to 2.39)^b^	0	.70
	Postmenopause	10 566	3	0.66 (0.42 to 1.03)	47.0	.15
All-cause mortality	Overall	14 114	3	0.95 (0.81 to 1.12)	0	.65
	Premenopause	3495	3	1.59 (1.11 to 2.26)^b^	0	.68
	Postmenopause	10 619	3	0.81 (0.61 to 1.09)	40.3	.19
Enterolactone						
Recurrence	Overall	3295	2	0.91 (0.67 to 1.23)	17.2	.27
Breast cancer–specific mortality	Overall	3864	3	0.72 (0.58 to 0.90)^a^	0	.57
	Prediagnosis	1457	1	0.74 (0.55 to 0.99)^a^	—^c^	—^c^
	Postdiagnosis	2407	2	0.70 (0.50 to 0.98)^a^	5.7	.30
	Premenopause	88	1	1.77 (0.46 to 6.84)	—^c^	—^c^
	Postmenopause	3776	3	0.66 (0.53 to 0.84)^a^	0	.49
All-cause mortality	Overall	3864	3	0.69 (0.57 to 0.83)^a^	0	.59
	Prediagnosis	1457	1	0.74 (0.59 to 0.93)^a^	—^c^	—^c^
	Postdiagnosis	2407	2	0.61 (0.45 to 0.83)^a^	0	.76
	Premenopause	88	1	1.85 (0.49 to 6.96)	—^c^	—^c^
	Postmenopause	3776	3	0.65 (0.51 to 0.82)^a^	22.7	.27
	Estrogen receptor positive	2652	2	0.75 (0.56 to 1.00)	0	.93
	Estrogen receptor negative	609	2	0.55 (0.28 to 1.08)	36.1	.21
	Node positive	721	2	0.84 (0.55 to 1.28)	0	.74
	Node negative	1583	2	0.41 (0.24 to 0.70)^a^	0	.42
Cruciferous vegetables						
Recurrence	Overall (pooled analysis)	11 390	1	1.09 (0.92 to 1.30)	—^c^	—^c^
Breast cancer–specific mortality	Overall	15 831	2	1.07 (0.91 to 1.26)	0	.59
All-cause mortality	Overall	17 041	3	0.99 (0.89 to 1.11)	0	.96
	Prediagnosis	1210	1	0.96 (0.64 to 1.43)	—^c^	—^c^
	Postdiagnosis	15 831	2	1.00 (0.89 to 1.12)	0	.83
Green tea						
Recurrence	Overall	1632	2	0.74 (0.55 to 1.01)	0	.68
	Stages I-II	1398	2	0.56 (0.38 to 0.83)^a^	0	.98
	Stage III-IV	234	2	1.32 (0.62 to 2.79)	25.7	.25

a Significant risk reductions.

b Significant increase in risk.

c Value not available (individual study result).

### Soy isoflavones

Soy isoflavone intake was associated with significantly reduced risk of breast cancer recurrence for the overall population, with moderate heterogeneity (HR = 0.74, 95% CI = 0.60 to 0.92; *I*^2^ = 58.3%; *P* = .7 for heterogeneity). After stratifying by menopause and estrogen receptor status, the effect remained significant only for the postmenopausal (HR = 0.72, 95% CI =  0.55 to 0.94; *I*^2^ = 45.8%; *P* = .16 for heterogeneity) and estrogen receptor–positive (HR = 0.82, 95% CI = 0.70 to 0.97) subgroups.

There was a nonsignificant risk reduction in breast cancer–specific mortality in the overall population (HR = 0.88, 95% CI = 0.75 to 1.04; *I*^2^ = 30.9%; *P* = .19 for heterogeneity). Results were similar when analyses were stratified according to estrogen receptor status: estrogen receptor–positive breast cancer (HR = 0.81, 95% CI = 0.65 to 1.02) and estrogen receptor–negative breast cancer (HR = 0.77, 95% CI = 0.60 to 0.98).

For all-cause mortality, nonsignificant risk reductions were found for the overall population (HR = 0.88, 95% CI = 0.77 to 0.997; *I*^2^ = 36.1%; *P* = .15 for heterogeneity) and in postmenopausal women (HR = 0.88, 95% CI = 0.76 to 1.02; *I*^2^ = 29.5%; *P* = .2 for heterogeneity). There was a significant reduction for stage III-IV breast cancer (HR = 0.58, 95% CI = 0.39 to 0.87). Stratification by tamoxifen use yielded similar results for users (HR = 0.81, 95% CI = 0.64 to 1.01) and nonusers (HR = 0.78, 95% CI = 0.62 to 0.98).

Dose-response analysis found evidence of nonlinearity for the association between soy isoflavones and breast cancer recurrence (*P* = .03 for nonlinearity), with a 30% reduction in hazard ratios at 60 mg/day but no further reduction in risk at higher intakes. Although the test for nonlinearity was not significant for soy isoflavones and breast cancer–specific mortality (*P* = .11 for nonlinearity) or all-cause mortality (*P* = .22 for nonlinearity), most of the reduction in risk was observed at the lower range of intake (20-40 mg/day) (Figure 3) (Supplementary Table 2, available online).

Results were similar when stratified by prediagnostic or postdiagnostic exposure assessment for all the overall endpoints. For soy isoflavones, the postdiagnostic subgroup combined data for exposure assessment time of less than and more than 12 months.

### Soy protein and products

Soy protein and/or products were investigated in the context of mortality in 4 studies (2 prediagnosis and 2 postdiagnosis). Soy product intake (two studies, 945 women) was associated with a significantly reduced risk of breast cancer recurrence for the overall population (HR = 0.48, 95% CI = 0.23 to 0.99; *I*^2^ = 25.4%; *P* = .25 for heterogeneity). For protein and products combined, there was no association with risk of breast cancer–specific or all-cause mortality (HR = 0.92, 95% CI = 0.76 to 1.11; *I*^2^ = 0%; *P* = .41 for heterogeneity and HR = 0.77, 95% CI = 0.49 to 1.21; *I*^2^ = 74.3%; *P* = .02 for heterogeneity, respectively). A significant risk reduction was found, however, for estrogen receptor–positive disease in breast cancer–specific mortality (HR = 0.75, 95% CI = 0.60 to 0.92; *I*^2^ = 0%; *P* = .9 for heterogeneity).

### Lignans

Lignans were investigated in 3 studies of prediagnosis intake. None investigated recurrence. There was no association with risk of breast cancer–specific or all-cause mortality (HR = 0.91, 95% CI = 0.74 to 1.12; *I*^2^ = 55.8%; *P* = .1 for heterogeneity and HR = 0.95, 95% CI = 0.81 to 1.12; *I*^2^ = 0%; *P* = .65 for heterogeneity, respectively). Increased risks were found in premenopausal women for both breast cancer–specific mortality (HR = 1.55, 95% CI = 1.01 to 2.39; *I*^2^ = 0%; *P* = .7 for heterogeneity) and all-cause mortality (HR = 1.59, 95% CI = 1.11 to 2.26; *I*^2^ = 0%; *P* = .68 for heterogeneity). In postmenopausal women, there were nonsignificant risk reductions for both endpoints: breast cancer–specific mortality (HR = 0.66, 95% CI = 0.42 to 1.03; *I*^2^ = 47%; *P* = .15 for heterogeneity) and all-cause mortality (HR = 0.81, 95% CI = 0.61 to 1.09; *I*^2^ = 40.3%; *P* = .19 for heterogeneity). The test for nonlinearity was not significant (Supplementary Table 3, available online).

### Enterolactone

**Figure 4.**
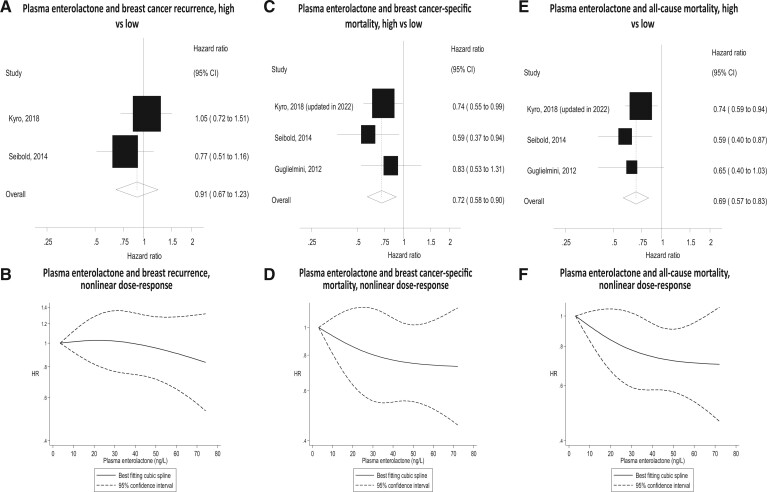
Forest plots for intake of serum/plasma enterolactone high vs low analyses and risk of recurrence, breast cancer–specific mortality, and all-cause mortality, with graphs illustrating nonlinear dose response. CI = confidence interval.

Serum and/or plasma ENL concentrations showed no association with recurrence in a meta-analysis of 2 studies (HR = 0.91, 95% CI = 0.67 to 1.23; *I*^2^ = 17.2%; *P* = .27 for heterogeneity). Significant inverse associations were found for breast cancer–specific mortality and all-cause mortality (HR = 0.72, 95% CI = 0.58 to 0.90; *I*^2^ = 0%; *P* = .57 for heterogeneity and HR = 0.69, 95% CI = 0.57 to 0.83; *I*^2^ = 0%; *P* = .59 for heterogeneity, respectively) (Figure 4). Stratification by menopause status showed that the risk reductions remained significant only for the postmenopause subgroup (HR = 0.66, 95% CI = 0.53 to 0.84; *I*^2^ = 0%; *P* = .49 for heterogeneity and HR = 0.65, 95% CI = 0.51 to 0.82; *I*^2^ = 22.7%; *P* = .27 for heterogeneity, respectively). Both the estrogen receptor–positive (HR = 0.75, 95% CI = 0.56 to 1.00; *I*^2^ = 0%; *P* = .93 for heterogeneity) and estrogen receptor–negative (HR = 0.55, 95% CI = 0.28 to 1.08; *I*^2^ = 36.1%; *P* = .21 for heterogeneity) subgroups showed nonsignificant risk reductions. For all-cause mortality, stratification by nodal status showed that the risk reduction remained significant for node-negative cancer only (HR = 0.41, 95% CI = 0.24 to 0.70; *I*^2^ = 0%; *P* = .42 for heterogeneity).

There was no evidence of nonlinearity for any endpoint (recurrence, *P* = .61 for nonlinearity; breast cancer–specific mortality, *P* = .68 for nonlinearity; all-cause mortality, *P* = .55 for nonlinearity) (Supplementary Table 4, available online).

Two ENL studies provided data for time periods up to 20 years: Guglielmini (53) found that the risk reductions for both breast cancer–specific and all-cause mortality lost significance by 10 years. Additional data provided by Kyrø (54), however, showed significant benefits at both 5 and 20 years for breast cancer–specific and all-cause mortality.

### Cruciferous vegetables

Cruciferous vegetables showed no effect according to meta-analyses on either breast cancer–specific mortality (HR = 1.07, 95% CI = 0.91 to 1.26; *I*^2^ = 0%; *P* = .59 for heterogeneity) or all-cause mortality (HR = 0.99, 95% CI = 0.89 to 1.11; *I*^2^ = 0%; *P* = .96 for heterogeneity) (Table 2) (Supplementary Table 5, available online). There was no effect on recurrence in 1 pooled analysis of 4 studies (57).

With the exception of 1 Asian study (SBCSS) in the pooled analysis (57), the median reported intake in the highest quantiles was less than 1 serving (78 g) per day.

### Green tea

**Figure 5.**
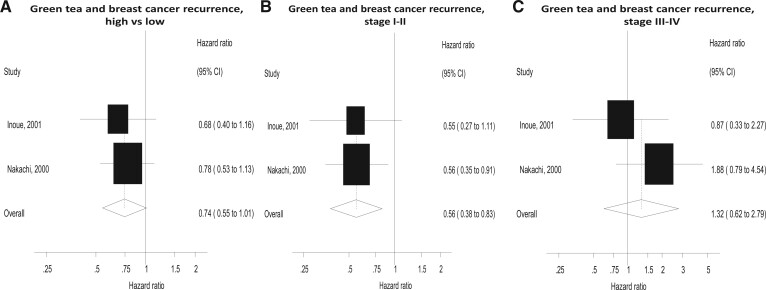
Forest plots for intake of green tea high vs low analyses and risk of breast cancer recurrence, overall and by stage. CI = confidence interval.

Green tea was investigated only in recurrence for prediagnostic intake (2 studies). A nonsignificant association was found for the overall population (HR = 0.74, 95% CI = 0.55 to 1.01; *I*^2^ = 0%; *P* = .98 for heterogeneity). Stratification by stage revealed a significant risk reduction in stage I and II breast cancer (HR = 0.56, 95% CI = 0.38 to 0.83) but not stage III or IV breast cancer (Figure 5).

### Sensitivity analysis

Only in the analysis of soy isoflavones was there a sufficient number of studies to conduct sensitivity analyses. For recurrence, excluding 1 study at a time, the summary hazard ratios ranged from 0.69 (95% CI = 0.59 to 0.80), when excluding the study by Ho et al. (44), to 0.82 (95% CI = 0.58 to 1.15), when excluding the pooled analysis by Nechuta et al. (47), suggesting that this result was driving the association (Supplementary Figure 3, available online).

In all-cause mortality, excluding 1 study at a time, the summary hazard ratios ranged from 0.85 (95% CI = 0.73 to 0.99), when excluding the study by Kyrø et al. (46), to 0.91 (95% CI = 0.83 to 1.00), when excluding the study by Fink et al. (43), but did not significantly affect the pooled estimate (HR = 0.88, 95% CI = 0.77 to 0.997).

### Modified GRADE assessment

**Figure 6.**
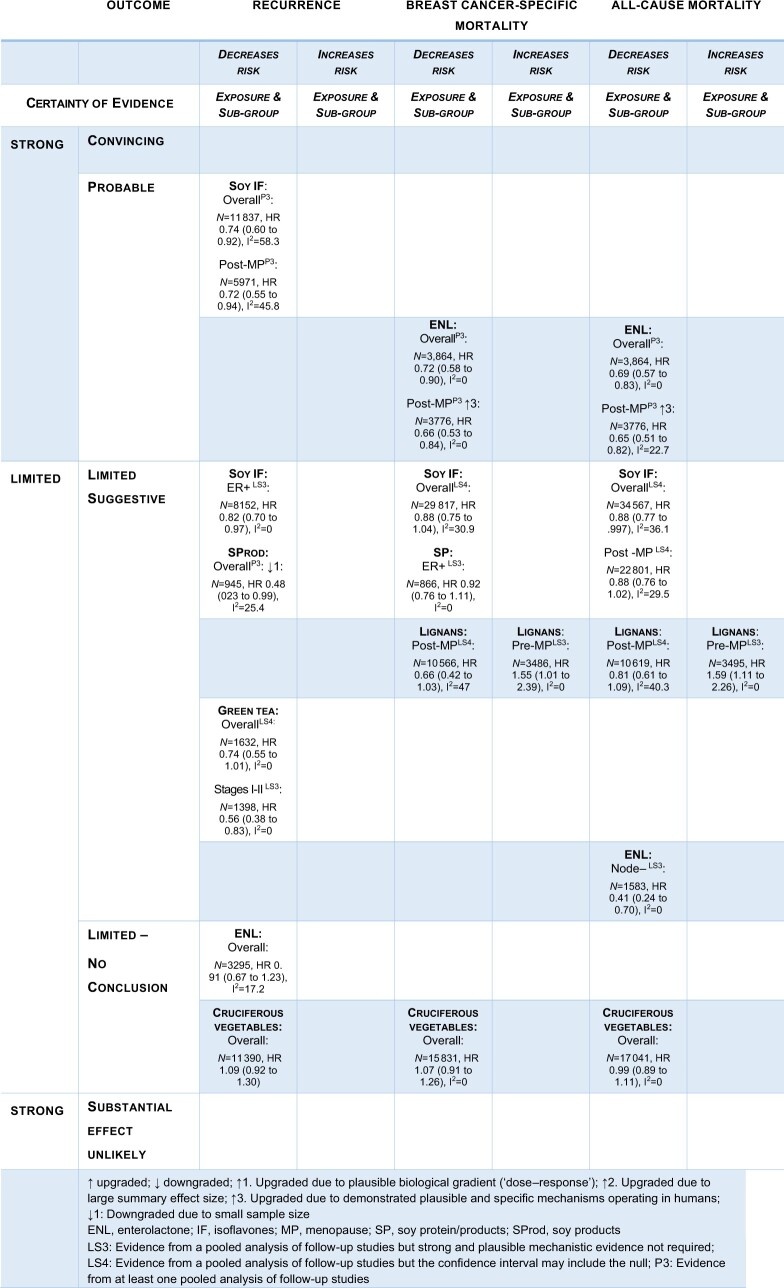
Evidence grading according to World Cancer Research Fund/American Institute for Cancer Research Adaptation of Grading of Recommendations, Assesment, Development, and Evolution criteria (26).

Certainty of evidence, graded according to the WCRF/AICR adaptation of GRADE for breast cancer survivors (26) (Figure 6) was probable for soy isoflavones in recurrence and serum and/or plasma ENL concentrations in breast cancer–specific and all-cause mortality, both overall and in the postmenopausal subgroups. Robust human experimental evidence exists for ENL.

The evidence for soy products in recurrence was downgraded to “limited suggestive” because of the small sample size. The evidence for lignans (prediagnosis) in breast cancer–specific and all-cause mortality was graded “limited suggestive” for premenopausal women as “strong and plausible mechanistic evidence was not required”; for results in postmenopausal women, “the confidence interval included the null.”

Green tea (prediagnosis) in recurrence for stage I and II breast cancer showed a large summary effect size (HR = 0.56, 95% CI = 0.38 to 0.83) but was graded as “limited suggestive” because of the small number of studies and the absence of robust human experimental evidence. No conclusion could be drawn for ENL in recurrence or cruciferous vegetables in any of the endpoints, but strong and plausible mechanistic evidence from preclinical studies exists for all exposures. No data were available for lignans in recurrence or green tea in mortality outcomes. Other subgroup findings were not graded because strong and plausible mechanisms have not been established.

## Discussion

The current systematic review and meta-analysis investigated the evidence from prospective and retrospective cohort studies for soy isoflavones, soy protein, lignans, ENL, cruciferous vegetables, or green tea in breast cancer survival and all-cause mortality. We particularly focused on the effective doses of these exposures and whether the evidence supports their initiation or substantial increase after diagnosis or cumulative prediagnostic intake.

### Soy

Soy isoflavones were associated with 26% reduced risk of recurrence, particularly among postmenopausal and estrogen receptor–positive survivors, with the greatest risk reduction at 60 mg/day and no further reduction at higher doses. This finding is consistent with the moderate intake of 2 to 3 servings (50-75 mg isoflavones) per day suggested as safe for women with breast cancer by the American Institute of Cancer Research (61) and the American Cancer Society (62,63). For mortality outcomes, the greatest reductions were at 20 to 40 mg/day (0.8-1.6 servings/day), which is consistent with daily intakes in Japan and Shanghai (64) but lower than current recommendations. One serving (25 mg isoflavones) can be provided by 250 mL soymilk, 85 to 100 g tofu, or 85 g cooked soybeans (64). Soy products were associated with a 52% risk reduction in recurrence (2 studies), and combined protein and products were associated with a 25% risk reduction in breast cancer–specific mortality for estrogen receptor–positive disease. These findings concur with the indications of links between eating soy 12 months or more after diagnosis and better breast cancer survival reported by WCRF/AICR (26).

### Lignans or enterolactone

For prediagnostic intake of dietary lignans, there were nonsignificant risk reductions for both cancer-specific and all-cause mortality in postmenopausal women (34% and 19%, respectively). In premenopausal women, however, for both endpoints, increased risks were seen. While this is suggestive of an interaction with the hormonal environment, the role of chance or residual confounders, such as treatment, cancer stage, and dietary change between recruitment and diagnosis cannot be ruled out. The highest mean lignan intake in the included studies was 9 mg/day or higher. Significant risk reductions were found for prediagnostic and early postdiagnostic serum and/or plasma ENL with breast cancer–specific (28%) and all-cause (31%) mortality for both time frames, particularly for postmenopausal women (34% and 35%, respectively). For all-cause mortality, there was a 59% reduction for node-negative disease. The greatest risk reduction for mortality outcomes was at the highest serum and/or plasma ENL levels, (80 ng/L). The lignan intake equivalent cannot be calculated from ENL data because of interindividual variability in bioactivation of lignans by the colonic microbiota (65).

### Green tea

The observed 44% risk reduction in recurrence for stage I and II breast cancer with prediagnostic intake of green tea was based on 2 studies that observed the greatest effect at 3 to 5 cups per day and 5 cups or more per day, respectively. According to WCRF/AICR modified GRADE criteria, these results suggest “probable” grade for soy isoflavones in recurrence and ENL in mortality outcomes but “limited suggestive” for green tea in estrogen receptor–positive disease for recurrence and other subgroup findings. Evidence of plausible biological and specific mechanisms actually operating in humans is strongest for ENL (66-71).

### Cruciferous vegetables

For cruciferous vegetables, no significant effects were found for any survival endpoints. It should be noted, however, that the intakes of cruciferous vegetables in the included studies do not reflect supplemental doses, which are typically at least an order of magnitude higher (72). For example, the recommended dose of I3C is approximately 300 to 400 mg/day, and the yield of I3C from glucosinolates is approximately 20% (73). Yet cooked cruciferous vegetables were found to contain no more than 60 mg glucosinolates per 100 g serving (74). Therefore, an effect on breast cancer progression at higher doses is still plausible.

It is important to note that published data have not captured the effect on survival outcomes of initiating these exposures following diagnosis because postdiagnosis dietary assessments include both established dietary habits and postdiagnostic dietary modification. No study provided results for introduction of the exposures at or following diagnosis.

Benefits for the phytoestrogen-containing exposures—soy and flaxseed—on overall survival outcomes could be attributable to their estrogen receptor–dependent and/or estrogen receptor–independent mechanisms. Soy isoflavones and the enterolignans from flaxseed and other sources, termed *phyto-SERMs,* have selective estrogen receptor–modulating activity and hence a selective pattern of estrogen agonist-antagonist activity (75). Soy isoflavones structurally resemble 17-β-estradiol, making them competitive inhibitors of estrogen at receptors in the breast. Genistein preferentially binds to estrogen receptorβ, with a 7- to 30-fold greater affinity for estrogen receptorβ than estrogen receptorα (76). Such xenobiotic estrogens are only weakly estrogenic, however, compared with endogenous estrogens (77,78). Thus, binding to estrogen receptors does not result in epithelial cell proliferation (75,79). The net estrogen agonist or antagonist activity may also depend on the hormonal milieu of the woman and could account for the differential associations observed with lignans according to menopausal status. Evidence for estrogen receptor–independent mechanisms from biomarker studies includes findings of an inverse association between genistein and Ki-67 at high levels of Ki-67 expression in invasive breast cancer (68), supporting an antiproliferative effect of soy isoflavones and significant reductions in proliferation (Ki-67 labelling index), HER2 expression, and an increase in apoptosis in breast cancer with 25 g/day flaxseed (69).

Concerns that soy could interfere with the activity of tamoxifen, potentially reducing or inhibiting its anticancer effects on breast tissue, are not supported by findings from clinical research, which suggests a benefit for recurrence and mortality in users (32,34,35,44,47) that was confined to postmenopausal women in 1 study (34). In the current study, results for tamoxifen user and nonuser subgroups were similar for both recurrence and all-cause mortality. The available data did not, however, permit stratification of exclusively postmenopausal women by tamoxifen use or nonuse. Therefore, it is not possible to distinguish the interaction of isoflavones with the estrogen environment from their interaction with tamoxifen.

Emerging evidence suggests that the gut microbiota and mammary microbiome may have a role in modulating the risk of breast cancer development and progression through several pathways, including metabolic pathways of estrogens, and that postmenopausal estrogen metabolism is associated with microbial diversity (80,81). There is considerable interindividual variation in gut metabolism of isoflavones (82) and lignans (83). Isoflavones are inherently estrogenic, although they may become further activated by conversion to equol, a bacterial metabolite of daidzein in the gastrointestinal tract (82). Only 30% to 50% of humans are equol producers (84). In the case of lignans, however, phytoestrogenic activity is completely dependent on colonic microbiota (85). In the present study, no association was found between lignans and mortality outcomes, but an inverse association was found for the lignan metabolite ENL, which may be attributable to the impact of the microbiota. The differential associations of lignans in mortality outcomes according to menopausal status cannot be wholly attributed to bioactivation by gut flora, however, because the effects for ENL were in the same direction. Although other components, such as soy protein or fiber or the unsaturated fatty acid α-linoleic acid in flaxseed, may be correlated with the measured biomarkers that could explain the associations, this study found that the effects of soy protein and products as well as trends for lignans were in the same direction as isoflavones and ENL, respectively, suggesting that these components contribute to the observed associations.

Evidence from studies in humans suggests several possible mechanisms for green tea catechins, including antiproliferative effects, demonstrated in a study of its effects on tumor Ki-67 in breast cancer tissue (86), and modest aromatase inhibition, but with no reports of interaction between green tea catechins and aromatase inhibitor drugs (87). Green tea catechins have demonstrated synergistic effects with conventional cancer treatments, reversing the tamoxifen-resistant phenotype in tamoxifen-resistant breast cancer; inhibiting the multidrug resistance P-glycoprotein activity, which is responsible for much of the resistance to chemotherapeutic drugs (87); and significantly augmenting the effectiveness of radiation therapy (88).

Differences between our results and those of previous studies are attributable to several factors. First, we include additional data from more recent studies, additional subgroup data from EPIC, and longer follow-up data from the Danish Diet and Health study. Second, we present separate analyses for soy isoflavones and soy protein as well as for lignans and ENL because of the critical role of colonic microbiota in bioactivation of lignans. Third, we identified inconsistencies in previous meta-analyses of soy and cruciferous vegetables in terms of classification of 1) the endpoint of DFS, 2) mortality outcomes, 3) prediagnosis and postdiagnosis time frames, 4) study inclusion, and 5) duplication of data (in some cases). We therefore contacted study authors for clarification. Novel findings of the current study were the significant risk reduction with soy isoflavones for recurrence in estrogen receptor–positive survivors and the significant associations between ENL and breast cancer–specific mortality as well as all-cause mortality for women with node-negative disease. Findings for the overall cohorts were otherwise consistent with those of previous meta-analyses examining high vs low intakes (17-19,21-23, 89,90).

### Strengths and limitations

Strengths of the current study include the comprehensive analyses of intake of these phytonutrients and their biomarkers in relation to survival outcomes, including high vs low analyses; dose-response analyses; and detailed subgroup, sensitivity, and influence analyses. We adapted the exposure ascertainment criterion for diet studies, refined the comparability criterion on the Newcastle-Ottawa Scale risk-of-bias tool, and graded the certainty of the evidence according the WCRF/AICR criteria for studies in breast cancer survivors (26). The overall quality of the included studies was high. Despite small study numbers, the total cohorts for each exposure were large. Additional data were sought from study authors and obtained for EPIC and the Danish Diet and Health study (46,54). Where necessary, authors were contacted for clarification of definitions used, study overlap, and prediagnosis and postdiagnosis exposure measurement, making this a more accurate, critical update of the evidence. The clinically relevant question of timing of initiation or increased intake of the exposures was explored.

The main limitation to determining the impact of exclusively postdiagnostic intake of these phytonutrients was the lack of stratification according to dietary modification at diagnosis. The small number of studies—and especially lack of consistency in reporting receptor subtypes and stage as well as different inclusion criteria—restricted the conduct of subgroup analyses.

Exposure ascertainment involved a range of food frequency questionnaires, not all of which were specifically validated for the exposure under investigation or included all diet sources and variabilities (91). Intake was measured at more than 1 time point in only a minority of studies (33,35,36,38,40,44).

Pooled analyses of soy (47) and cruciferous vegetables (57) were included in preference to the individual studies to capture data from unpublished studies, although this meant excluding 2 other cruciferous vegetable studies: Nurses Health Study II (NHSII) (33), which found a significant inverse association for all-cause mortality, and a subpopulation of tamoxifen users from Women’s Healthy Eating and Living (WHEL), with a larger sample size (36), which found a nonsignificant risk reduction for recurrence among tamoxifen users.

In the analyses including soy products, the greatest contribution was from a study of exclusively fermented soy products in both the recurrence and all-cause mortality endpoints (51). Thus, the effects from other factors fermentation produces cannot be ruled out.

### Recommendations

Greater consistency among researchers designing observational studies is needed to permit meta-analyses and meaningful comparisons of studies that would assist clinicians in advising patients of the risks and benefits of phytonutrients in breast cancer. Five areas are candidates for such consensus:

1. **Definitions of endpoints.** The authors propose the following definitions:
  - *Recurrence.* The “time from diagnosis to a second breast cancer event, including ipsilateral, contralateral, regional, distant recurrence as well as new breast primaries; deaths are censored”
  - *Breast cancer–specific mortality.* The “time from diagnosis to death due to breast cancer; other deaths are censored”
  - *Disease-free survival.* The “time to recurrence of tumor or breast cancer–specific mortality”
  - *Distant disease-free survival.* The “time to distant recurrence and death from any cause”
    It is suggested that local and distant recurrence be separated because they have different disease progression and prognoses.
2. **Stratification.** Stratification according to menopause status as well as current endocrinological treatment is important in studies of phytoestrogens because of their potentially differential effects according to the hormonal milieu. Former use of endocrine therapy should be examined separately from concurrent use.
  For all exposures, stratification according to stage and hormone receptor status is needed—at a minimum, estrogen receptor positivity and negativity but preferably by molecular subtypes (luminal a, luminal b, HER2 positivity, and triple negative)—because the main forms of treatment as well as prognoses vary according to the molecular subtype.
  Because of the potentiating effect of phytoestrogens and green tea catechins on chemotherapy and radiation therapy, sensitivity analysis is needed for intake of these exposures with concurrent, adjuvant, and neoadjuvant treatment.
3. **Exposure ascertainment.** Food frequency questionnaires should be specifically validated for the exposure under investigation.
  Controlling for antibiotic use is recommended in studies of dietary lignans because of its impact on modulating their bioactivation (83). Although observational studies assume that the Cox model association is the same throughout follow-up, despite its length, dietary intake or its impact may not remain constant over that time. Postdiagnosis dietary modification is common in patients with breast cancer but not necessarily maintained long term (92-94). Therefore, frequently repeated exposure measurements are needed to capture changes over time.
4. **Dose.** In studies of green tea, information about EGCG content per cup (59) and grams per cup should be reported to help establish phytoequivalence. Examining the shape of the dose-response relationship between the exposures and outcomes using cubic splines is recommended.
5. **Exposure time frame.** Postdiagnostic exposure assessment should optimally be 1 year after diagnosis. Data should be presented separately for measurement within the first 12 months of diagnosis, when fluctuations resulting from tumor burden and chemotherapy exposure make the assessment less reliable. Measuring too long after diagnosis increases the risk of reverse-causality.

Data for exclusively postdiagnostic intake is needed to determine the impact of initiating an exposure or increasing its intake following diagnosis on survival outcomes. Therefore, changes in intake at or after the time of diagnosis need to be measured and compared with exclusively prediagnostic and pre- plus postdiagnostic intake.

### Other

To address the issue of the interindividual variability of the microbiota in bioactivation of lignans, dietary intake assessment could be complemented with biomarker measurements using appropriate and validated analytical methods (91), although some validation studies of food frequency questionnaires have found good correlation with biomarkers studies for soy and lignans (95,96). The relevance of the gut microbiome, however, may not be confined to lignan studies. Evidence of its impact on breast cancer progression suggests a possible future role for including interventions that enhance specific gut bacterial species (81).

To distinguish the interaction of phytoestrogens with the estrogen environment from their interaction with endocrine therapy, further evidence is needed from large studies that stratify according to menopausal status and endocrine treatments.

This meta-analysis was the first to demonstrate risk reductions for soy isoflavones in recurrence in women with estrogen receptor–positive breast cancer and for ENL in breast cancer–specific mortality and node-negative survivors in all-cause mortality. The magnitude of the effects was up to 59%. Dosages for soy were consistent with current recommendations for recurrence but lower than current recommendations for mortality outcomes. Findings regarding the impact of prediagnostic intake are important to inform nutritional guidelines. The potential differential effects of prediagnostic lignan intake according to menopause status warrants further investigation. To further inform clinical practice, evidence is required on the impact of dietary and supplemental intakes of phytonutrients introduced or substantially increased following diagnosis and treatment.

## Supplementary Material

pkad104_Supplementary_Data

## Data Availability

Additional data analyzed for this article were provided by Cecilie Kyrø by permission. Data will be shared upon application to the corresponding author, with approval of Cecilie Kyrø and the relevant authorities.
