# Supplementary material for: Phytonutrients and outcomes following breast cancer: a systematic review and meta-analysis of observational studies
Source: JNCI Cancer Spectr. 2023 Dec 9;8(1):pkad104. doi: 10.1093/jncics/pkad104 (PMC10868383; doi:10.1093/jncics/pkad104)
Supplement: pkad104_Supplementary_Data [file pkad104_supplementary_data.pdf]

Supplementary Table 1 Quality and risk of bias assessment on modified Newcastle-Ottawa Scale

First author, year	S1: Exposed cohort representative	S2: Selection of low dose/non-exposed	S3: Exposure ascertainment	S4: Demonstrated outcome of interest	Total selection score:	C1: Controlled for specified primary	C2: Controlled for additional	Total comparability score:	O1: Assessment of outcome	O2: Follow-up long enough for outcome	O3: Adequacy of follow-up of cohort	Total outcome score:	Total NOS score
Caan, 2011	1	1	1	1	4	1	1	2	1	1	1	3	9
Guha, 2009	1	1	1	1	4	1	0	1	1	1	1	3	8
Shu, 2009	1	1	1	1	4	1	1	2	1	1	1	3	9
Nechuta, 2012	1	1	1	1	4	1	1	2	0	1	1	2	8
Fink, 2007	1	1	1	1	4	1	0	1	1	1	1	3	8
Boyapati, 2005	1	1	1	1	4	1	0	1	1	1	1	3	8
Kang, 2010	1	1	1	0	3	1	1	2	1	1	1	3	8
Zhang, 2012	1	1	0	0	2	1	0	1	0	1	1	2	5
Kang, 2012	1	1	1	0	3	1	0	1	0	1	1	2	6
Conroy, 2013	1	1	1	0	3	1	1	2	1	1	1	3	8
Kyrø, 2015	1	1	0	1	3	1	0	1	1	1	1	3	7
Zhang, 2017	1	1	1	1	4	1	0	1	1	1	1	3	8
Ho, 2021	1	1	1	0	3	1	1	2	1	1	1	3	8
Woo, 2012	1	1	1	1	4	1	0	1	1	1	1	3	8
Yang, 2023	1	1	1	1	4	1	0	1	0	1	1	2	7
McCann, 2010	1	1	1	1	4	1	0	1	1	1	1	3	8
Guglielmini 2012	1	1	1	1	4	1	0	1	1	1	1	3	8
Seibold, 2014	1	1	1	1	4	1	1	2	1	1	1	3	9
Buck, 2011a	1	1	1	1	4	1	1	2	1	1	1	3	9
Buck, 2011b	1	1	1	1	4	1	1	2	1	1	1	3	9
Olsen, 2011	1	1	1	1	4	1	0	1	1	1	1	3	8

Kyrø, 2018	1	1	1	1	4	1	1	2	1	1	1	3	9
Jaskulski, 2018	1	1	1	1	4	1	1	2	1	1	1	3	9
Jaskulski, 2020	1	1	1	1	4	1	1	2	1	1	1	3	9
Nechuta, 2013	1	1	1	0	3	1	1	2	1	1	1	3	8
Fink 2006	1	1	1	1	4	1	0	1	1	1	1	3	8
Beasley, 2011	1	1	0	1	3	1	0	1	1	1	1	3	7
Farvid, 2020	0	1	1	0	2	1	1	2	1	1	0	2	6
Thomson, 2011	1	1	1	1	4	1	0	1	1	1	1	3	8
Nakachi, 2000	1	1	1	1	4	1	1	2	0	1	0	1	7
Inoue, 2001	1	1	1	1	4	1	0	1	1	1	1	3	8
Bao, 2015	1	1	1	1	4	1	1	2	0	1	1	2	8

Supplementary Table 2 Nonlinear dose-response analysis of soy isoflavones and breast cancer survival outcomes

Recurrence		Breast-cancer-specific mortality		All-cause mortality	
mg/d	HR (95% CI)	mg/d	HR (95% CI)	mg/d	HR (95% CI)
1.54	1.00	0.1	1.00	0.1	1.00
10	0.89 (0.81-0.97)	10	0.86 (0.75-0.99)	10	0.87 (0.72-1.06)
20	0.80 (0.67-0.95)	20	0.78 (0.63-0.98)	20	0.80 (0.59-1.08)
30	0.74 (0.60-0.93)	30	0.75 (0.56-1.01)	30	0.77 (0.55-1.08)
40	0.72 (0.56-0.92)	40	0.73 (0.51-1.07)	40	0.77 (0.55-1.06)
50	0.71 (0.55-0.91)	50	0.73 (0.46-1.17)	50	0.78 (0.58-1.04)
60	0.70 (0.55-0.90)	60	0.73 (0.41-1.30)	60	0.79 (0.61-1.02)
70	0.71 (0.55-0.90)	70	0.73 (0.37-1.46)	70	0.80 (0.63-1.01)
80	0.71 (0.55-0.91)	80	0.73 (0.33-1.64)	80	0.81 (0.64-1.02)
90	0.71 (0.55-0.91)	90	0.73 (0.29-1.85)	90	0.82 (0.64-1.05)
100	0.71 (0.54-0.92)	100	0.73 (0.26-2.09)	100	0.83 (0.64-1.09)
$p_{non-linearity}$	0.03	$p_{non-linearity}$	0.11	$p_{non-linearity}$	0.22

Supplementary Table 3 Nonlinear dose-response analysis of dietary lignans and breast cancer survival outcomes

Breast-cancer-specific mortality		All-cause mortality	
mg/d	HR (95% CI)	mg/d	HR (95% CI)
0.1	1.00	0.1	1.00
1	0.98 (0.63-1.53)	1	0.97 (0.70-1.34)

2	0.96 (0.46-2.03)	2	0.95 (0.55-1.65)
3	0.93 (0.38-2.31)	3	0.94 (0.49-1.82)
4	0.90 (0.33-2.41)	4	0.94 (0.47-1.87)
5	0.86 (0.30-2.45)	5	0.94 (0.48-1.87)
6	0.83 (0.27-2.52)	6	0.95 (0.48-1.87)
7	0.79 (0.24-2.64)	7	0.95 (0.48-1.86)
8	0.76 (0.21-2.81)	8	0.95 (0.48-1.87)
9	0.73 (0.18-3.02)	9	0.95 (0.48-1.88)
10	0.70 (0.15-3.27)	10	0.96 (0.48-1.89)
$p_{non-linearity}$	0.92	$p_{non-linearity}$	0.86

Supplementary Table 4 Nonlinear dose-response analysis of serum/plasma enterolactone and breast cancer survival outcomes

Recurrence		Breast-cancer-specific mortality		All-cause mortality	
ng/L	HR (95% CI)	ng/L	HR (95% CI)	ng/L	HR (95% CI)
3.5	1.00	3.1	1.00	3.1	1.00
10	1.02 (0.88-1.19)	10	0.91 (0.73-1.12)	10	0.89 (0.76-1.05)
20	1.03 (0.78-1.37)	20	0.83 (0.57-1.21)	20	0.81 (0.61-1.07)
30	1.03 (0.73-1.44)	30	0.78 (0.50-1.21)	30	0.75 (0.55-1.04)
40	1.00 (0.71-1.41)	40	0.75 (0.49-1.12)	40	0.72 (0.53-0.96)
50	0.96 (0.69-1.33)	50	0.73 (0.52-1.02)	50	0.70 (0.54-0.89)
60	0.91 (0.65-1.28)	60	0.72 (0.52-0.98)	60	0.68 (0.53-0.88)

70	0.86 (0.58-1.29)	70	0.71 (0.47-1.07)	70	0.68 (0.47-0.98)
$p_{non-linearity}$	0.61	$p_{non-linearity}$	0.68	$p_{non-linearity}$	0.55

Supplementary Table 5 Nonlinear dose-response analysis of cruciferous vegetables and breast cancer survival outcomes

Breast-cancer-specific mortality		All-cause mortality	
g/d	HR (95% CI)	g/d	HR (95% CI)
7.8	1.00	5.7	1.00
10	1.04 (0.95-1.13)	10	1.02 (0.96-1.08)
20	1.08 (0.91-1.28)	20	1.03 (0.92-1.16)
30	1.11 (0.87-1.41)	30	1.05 (0.89-1.23)
40	1.13 (0.85-1.51)	40	1.05 (0.87-1.28)
50	1.15 (0.84-1.57)	50	1.06 (0.86-1.29)
60	1.15 (0.84-1.59)	60	1.05 (0.86-1.29)
70	1.16 (0.84-1.59)	70	1.05 (0.87-1.27)
80	1.15 (0.85-1.57)	80	1.05 (0.87-1.26)
90	1.15 (0.86-1.54)	90	1.04 (0.897-1.25)
$p_{non-linearity}$	0.46	$p_{non-linearity}$	0.58

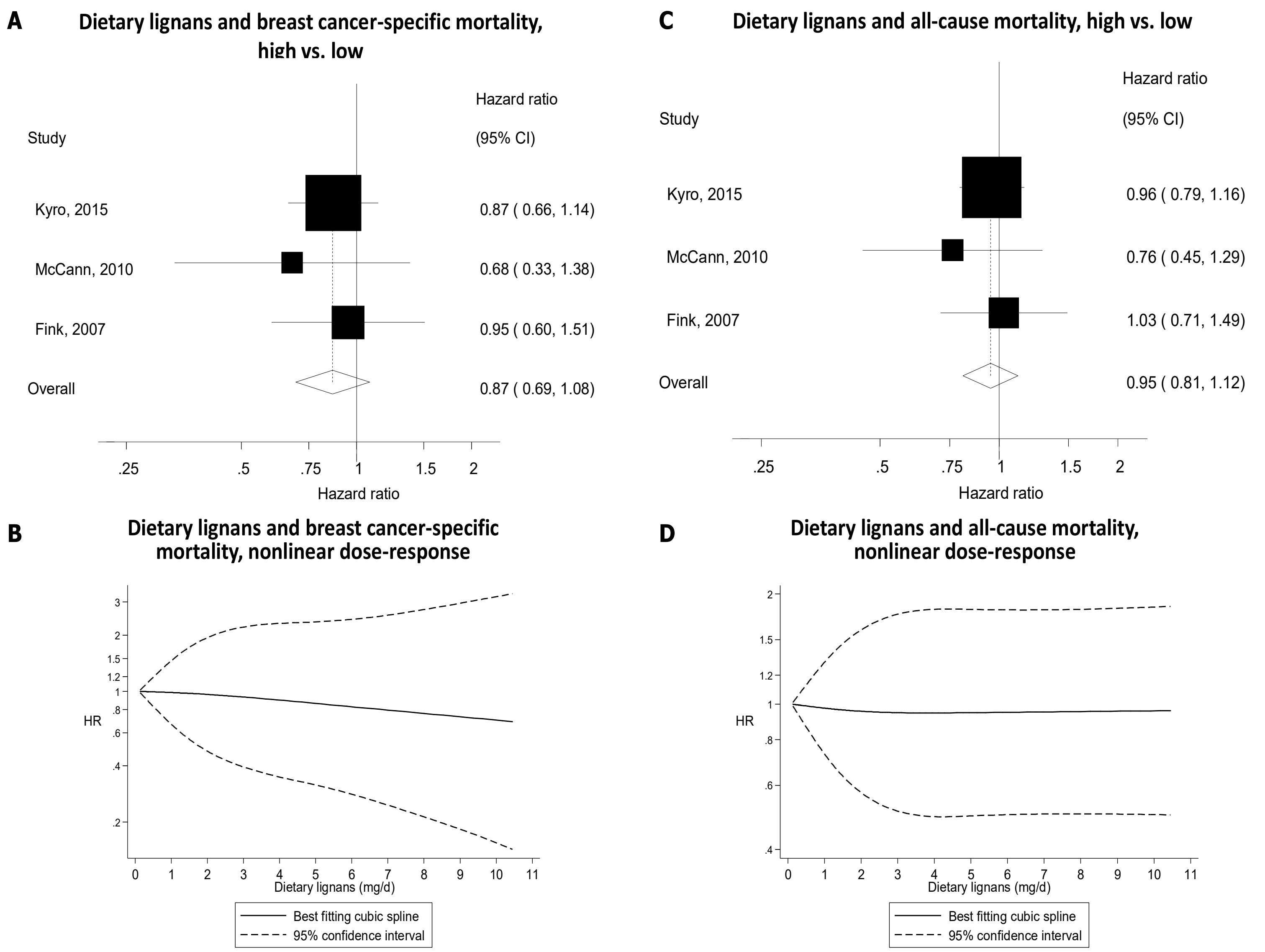

Supplementary Figure 1: Forest plots for intake of lignans high versus low analyses and risk of breast cancer recurrence, breast cancer-specific mortality and all-cause mortality, with graphs illustrating non-linear dose-response

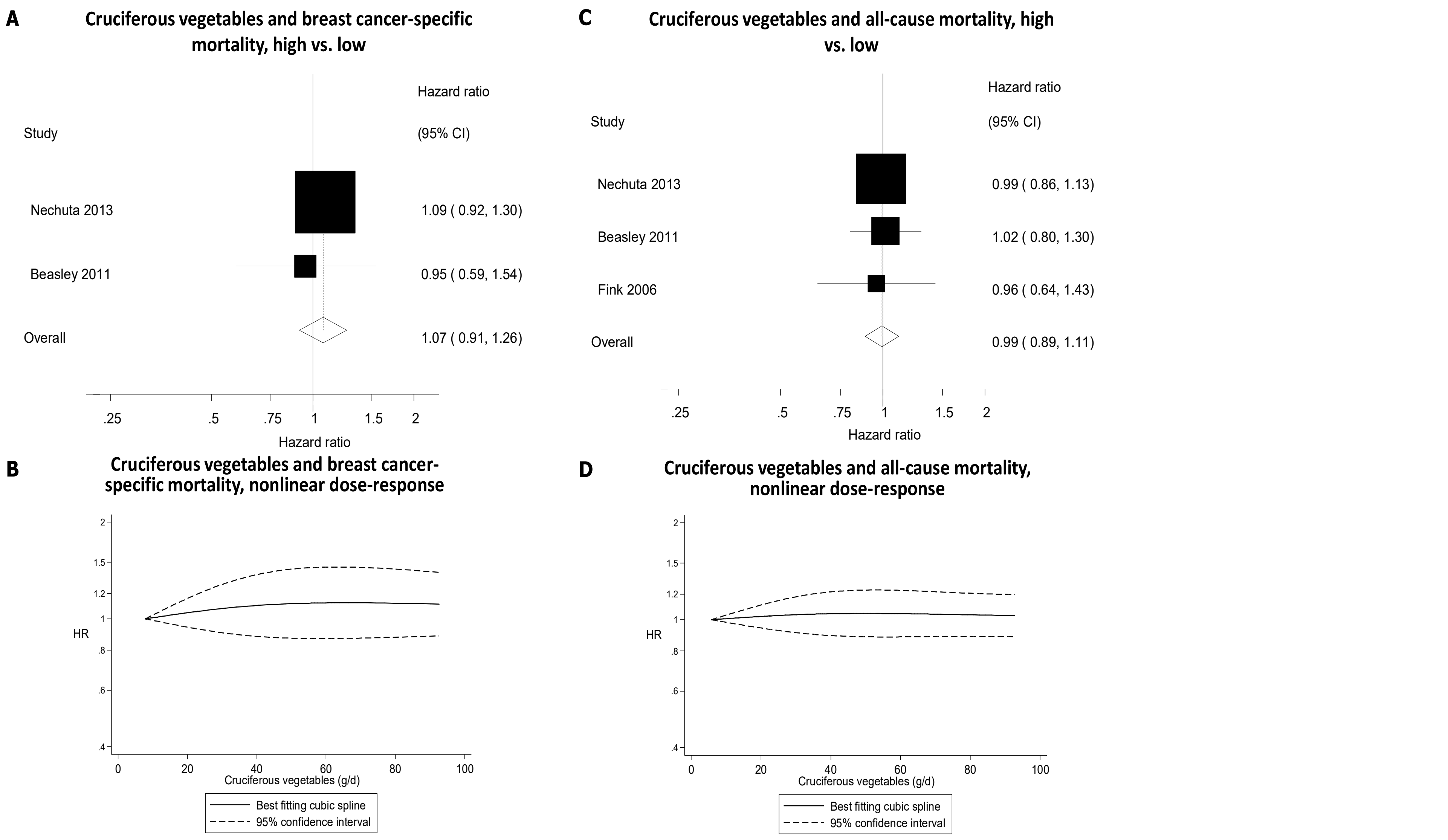

Supplementary Figure 2: Forest plots for intake of cruciferous vegetables high versus low analyses and risk of breast cancer recurrence, breast cancer-specific mortality and all-cause mortality, with graphs illustrating non-linear dose-response

a. Recurrence

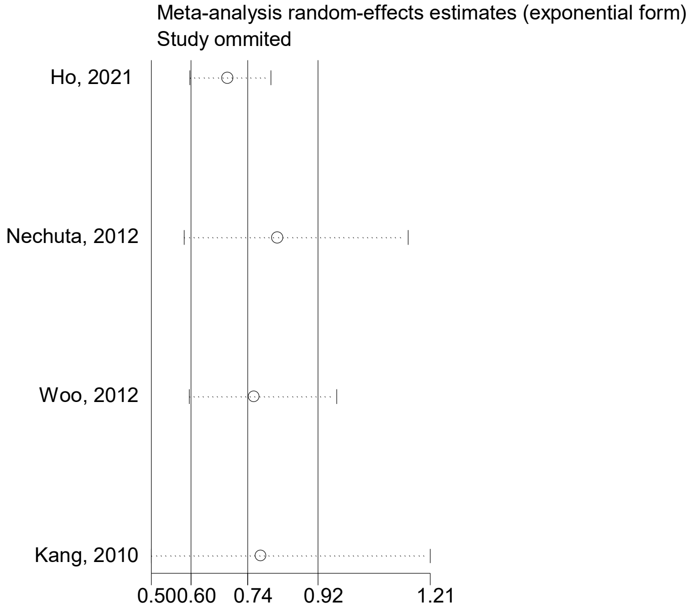

Study omitted	e^coef.	[95% Conf.	Interval]
Ho, 2021	0.68962014	0.59399378	0.80064124
Nechuta, 2012	0.81694788	0.58018762	1.1503242
Woo, 2012	0.7574411	0.59211063	0.96893543
Kang, 2010	0.77422225	0.49646565	1.2073747
Combined	0.74157663	0.59689718	0.92132434

b. Breast cancer-specific mortality

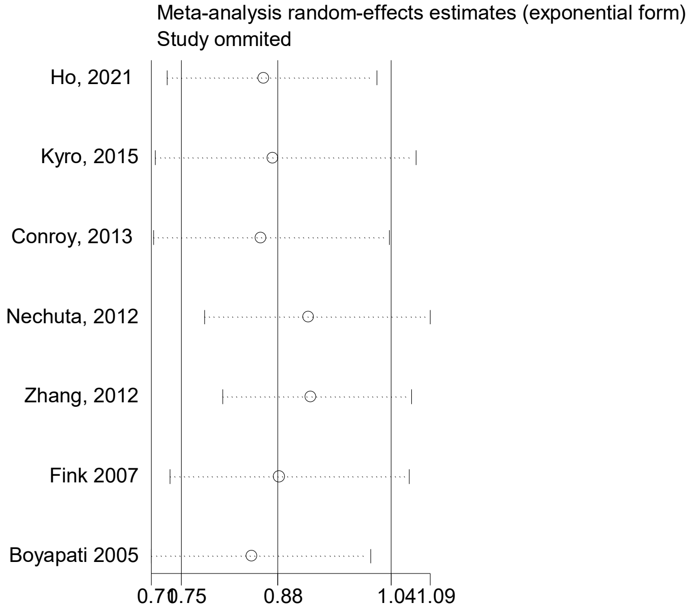

Study omitted	e^coef.	[95% Conf. Interval]	
Ho, 2021	0.86360025	0.7330606	1.0173857
Kyro, 2015	0.87589234	0.71678215	1.0703214
Conroy, 2013	0.85940588	0.71403396	1.0343744
Nechuta, 2012	0.92380369	0.78326935	1.0895528
Zhang, 2012	0.92707086	0.80772734	1.0640477
Fink 2007	0.884148	0.73662162	1.0612202
Boyapati 2005	0.84727395	0.7115609	1.008871
Combined	0.88285381	0.75210681	1.03633

c. All-cause mortality

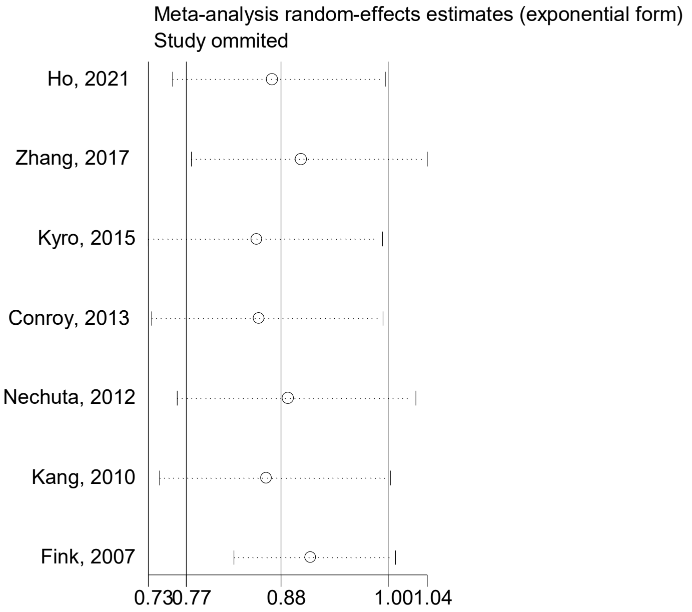

Study omitted	e^coef.	[95% Conf. Interval]	
Ho, 2021	0.86843401	0.75861913	0.99414527
Zhang, 2017	0.90027058	0.77901298	1.0404025
Kyro, 2015	0.85160452	0.73184401	0.99096286
Conroy, 2013	0.85384554	0.73517835	0.99166709
Nechuta, 2012	0.88579416	0.76364309	1.0274843
Kang, 2010	0.86227417	0.74380189	0.99961674
Fink, 2007	0.91122103	0.82617182	1.0050256
Combined	0.87842724	0.77384353	0.99714528

Supplementary Figure 3: Influence analysis for high vs. low soy isoflavones and (a) recurrence; (b) breast cancer-specific mortality; (c) all-cause mortality
